# TG1042 (Adenovirus-interferon-γ) in Primary Cutaneous B-cell Lymphomas: A Phase II Clinical Trial

**DOI:** 10.1371/journal.pone.0083670

**Published:** 2014-02-24

**Authors:** Brigitte Dreno, Mirjana Urosevic-Maiwald, Youn Kim, Joan Guitart, Madeleine Duvic, Olivier Dereure, Amir Khammari, Anne-Chantal Knol, Anna Derbij, Monika Lusky, Isabelle Didillon, Anne-Marie Santoni, Bruce Acres, Vincent Bataille, Marie-Pierre Chenard, Pascal Bleuzen, Jean-Marc Limacher, Reinhard Dummer

**Affiliations:** 1 Department of Skin Cancer, University Hospital, Nantes, France; 2 Department of Dermatology, University Hospital, Zurich, Switzerland; 3 Stanford University School of Medicine, Stanford, California, United States of America; 4 Northwestern University Medical School, Chicago, Illinois, United States of America; 5 University of Texas, M.D. Anderson Cancer Center, Houston, Texas, United States of America; 6 Department of Dermatology, Saint Eloi Hospital, Montpellier, France; 7 Transgene, Illkirch (Strasbourg), France; 8 Department of Pathology, University Hospital, Strasbourg, France; National Cancer Institute, United States of America

## Abstract

**Rational:**

While a variety of registered therapies exist for Cutaneous T Cell Lymphoma, no such therapy is available for Cutaneous B Cell Therapy. In this context we performed a phase II, open label, multicenter, non-comparative study to evaluate the efficacy and safety of repeated intra-lesional administrations of TG1042 (adenovirus-interferon-γ) in patients with relapsing primary cutaneous B-cell lymphomas (CBCL).

**Method:**

Thirteen patients have been enrolled and received intralesional injections of TG1042 containing 5×10^10^ viral particles into up to six lesions simultaneously. Injections were performed on days 1, 8 and 15 of each of four consecutive 28 day cycles.

**Results:**

Eleven (85%) out of 13 enrolled patients showed an objective response after injections of TG1042. Seven patients (54%) exhibited complete and four (31%) displayed partial response. The median time to disease progression in the study population was 23.5 months (range 6.25 to 26+). Most commonly observed adverse events were minor to moderate flu-like symptoms, fatigue and injection site reactions.

**Conclusions:**

Our study showed that treatment with TG1042 was associated with a clinical benefit in the majority of the patients with relapsing CBCL, including tumor regression, a clinically meaningful duration of response and a good treatment tolerance.

**Trial Registration:**

www.clinicaltrials.gov NCT00394693

## Introduction

Cutaneous lymphomas (CL) represent a heterogeneous group of Non-Hodgkin's lymphomas and are the second most frequent extranodal lymphomas [1]. They are characterized by lymphoproliferative clonal infiltrates of mainly T- or B-cell initially appearing in the skin [2], [3], [4]. During progression of the disease, the lesions can change shape, eventually forming plaques and tumors before developing extracutaneous disease. The most common type of cutaneous lymphoma is the cutaneous T-cell lymphoma (CTCL). Primary cutaneous B-cell lymphomas (CBCLs) with an incidence rate of approximately 3.1 per 1′000′000 persons and per year represent 20–25% of all cutaneous lymphomas [5], [6]. The most common CBCL subtypes are: primary cutaneous follicular lymphoma (PCFL), primary cutaneous marginal zone lymphoma (PCMZL), and diffuse large B-cell lymphoma, leg-type [4].

While a variety of registered therapies exist for CTCL, no such therapy is available for CBCL. For the management of relapsing CBCL, after first line radiotherapy or surgery, various off-label therapeutic options are being explored. Curative treatments include radiotherapy and surgery, but disease relapses justify to seek alternative therapeutics [7]. Immunotherapeutic approaches, such as recombinant IFN-γ and IFN-α have shown clinical efficacy in this group of diseases. Systemic administration of IFN-γ has shown promising response rates in CTCL, however, therapy with recombinant cytokines is limited by their short half-life and significant side effects upon systemic administration [8].

TG1042 is an adenovirus 5 expressing the cDNA of human IFN-γ gene. In the previously conducted phase I/II, open label, multicenter, dose-escalation study, 38 CTCL and CBCL patients received intra-tumoral injections of TG1042. Local tumor response could be observed in all 5 treated CBCL patients, with 3 complete responses and 2 partial responses. TG1042 was well tolerated with minor to moderate injection side reactions and flu-like symptoms as most frequent adverse events [9], [10].

Considering clinical responses in CBCL patients and absence of approved medicinal therapy in this indication, we conducted a multicenter phase II study in patients with relapsing primary CBCL to evaluate the efficacy and safety of intra-lesional administrations of TG1042 in this population of patients. Our results show high response rates of CBCL to intra-lesional TG1042 treatment as well as good tolerability making this gene-therapy based immunotherapy a reasonable option for these cutaneous lymphomas.

## Materials and Methods

### Study design and patients

The protocol for this trial and supporting checklist are available as supporting information; see Checklist S1 and Protocol S1. This open label single arm study was approved by the local institutional ethical committees and the national authorities for biosafety in Switzerland, France and USA, and conducted in accordance with the ethical principles of the Declaration of Helsinki, in compliance with the approved protocol and its amendments, and good clinical practice principles. The study obtained approval from national health agencies in all countries where it took place and from the respective ethics committees or institutional review boards for all participating centers (University Hospital, Nantes, France; University Hospital, Zurich, Switzerland; Stanford University School of Medicine, California, USA; Northwestern University Medical School, Chicago, Illinois, USA; M.D. Anderson Cancer Center, Houston, Texas, USA; Saint Eloi Hospital, Montpellier, France). The study was conducted in accordance with the ethical principles of the Helsinki Declaration, the International Conference of Harmonization Good Clinical Practice guidelines and the local regulatory requirements. Written informed consent was obtained from each patient. The clinical trial was registered under ClinicalTrials.gov with the identifier NCT00394693. To be eligible for the study, the selected patients had to fulfill the following most important inclusion criteria: histologically confirmed diagnosis of primary CBCL, including (according to WHO/EORTC classification 2005 [4]) primary cutaneous marginal zone B-cell lymphoma, primary cutaneous follicle center B-cell lymphoma, primary cutaneous diffuse large B-cell lymphoma other than leg type (for all countries except in France, as a request from local authorities) and T-cell/histiocyte-rich B-cell lymphoma; relapse or active disease after at least one first line of treatment by radiotherapy or other standard therapy. Patients with primary cutaneous diffuse large B-cell lymphoma, leg-type, were excluded. All prior treatments for CBCL had to be stopped 4 weeks prior to patient enrollment into the study. Disease progression requiring other forms of specific antitumor therapy during the study was a cause for discontinuation from the study. An independent pathology review of the biopsy samples obtained before and after treatment was performed. No discrepancy was found between initial diagnosis given by the investigator and the one that was given by the independent pathology review.

### Study drug, administration procedure

Patients received from the participating investigators intralesional injections of TG1042 at the dose of 5×10^10^ viral particles (vp) per lesion into up to six lesions treated simultaneously on days 1, 8 and 15. Patients received no treatment during the fourth week of the cycle. The cycle length was 4 weeks (1 cycle), at the end of the cycle the tumors were evaluated for response (Day 29). In the absence of progression an additional cycle was to be delivered up to a maximum of four cycles. In the event of local response and consequent disappearance of the initially-treated lesion(s), the investigator was to decide if applicable whether or not to treat a second series of one or several additional lesion(s) that were not previously treated.

### Tumor response, quality of life and safety evaluations

The primary objective of the study was the response rate of the treated lesions in the group of study participants. The response to the treatment was defined in a given patient as the best objective tumor response, which was recorded from the first cycle evaluation until the last follow up visit on study. The response was to be confirmed by a repeated assessment performed no less than 4 weeks after the criteria for response were first met. Complete response (CR) was defined as the clinical disappearance of all treated lesions. Partial response (PR) was defined as the clinical disappearance of at least half of all treated lesions. Minor response (MR) was defined as a decrease of at least 50% in the sum of the products of all treated lesions with the clinical disappearance of less than half of all treated lesions. Progressive disease (PD) was defined as an increase of at least 25% in the sum of the products of all treated lesions. Stable disease (SD) was defined as any response that did not meet the criteria for CR, PR, MR or PD. Total response assessment used the same definitions applied to all the lesions but also integrated for the definition of PD the appearance of new cutaneous lesions, lymph nodes, hematological or visceral involvement.

An evaluation of all lesions (treated and not treated) was performed at baseline and on Day 29 of each cycle, at the last treatment cycle visit and at each follow-up visit or up to tumor progression. The treatment efficacy in patients that had shown a CR as best objective tumor response was confirmed by pathological analysis of skin biopsies at the site of the treated lesions.

An independent histo-pathology review was performed on the biopsies obtained from the 13 patients, in order to confirm the initial diagnosis of primary cutaneous B-cell lymphomas and the treatment efficacy of TG1042 for patients who achieved a CR. Secondary endpoints included the assessment of safety as well as quality of life

The condition of the patient was monitored for adverse events (AEs) throughout the study by clinical safety assessments by the investigator. The intensity of AEs was graded according to the NCI-CTCAE version 3.0 (see the website http://ctep.cancer.gov/protocolDevelopment/electronic_applications/docs/ctcaev3.pdf (accessed January 14th 2013)).

All patients were evaluated for quality of life using the Dermatology Life Quality Index (DLQI) questionnaire at each visit. The DLQI questionnaire allows a scoring from 0 to 30 corresponding respectively to no effect at all on patient's life and an extremely large effect on patient's life [11]. A post-treatment follow-up was performed on a monthly basis for the first 6 months and then on a quarterly basis for one year or until progression.

### Statistical design and analyses

The purpose of the trial was to reject the experimental treatment from being further studied, if TG1042 was shown to be insufficiently active in the considered indication and to accept it for further studying, if it was shown to be active. The study was designed as a two-stage ‘optimum’ phase II trial with the following assumptions: the inactivity cut-off was chosen equal to 50%, the activity cut-off equal to 75%.

Hence the hypotheses of interest were H_0_: r≤50% against H_A_: r≥75%, where r is the response rate. The type I error rate (α, probability of accepting an insufficiently active treatment, a false positive outcome) was set to 5%. The type II error rate (β, probability of rejecting an active treatment, a false negative outcome) was set to 10%. Under these assumptions, the first step of the optimal design consisted in treating 13 evaluable patients, if at most 7 responses were observed, the trial was to be stopped and the drug declared insufficiently active. If at least 8 responses were observed, the drug was to be declared active and additional patients were to be treated up to a total of 41 evaluable patients with the aim of obtaining a total of 26 responses to confirm TG1042 is active. After the first step of the study achieved its endpoint and showed a sufficient level of activity, the sponsor decided to not initiate the second step of the statistical design for company reasons.

The Intent-To-Treat population (ITT) (N = 13) included all patients enrolled in the study including the one non-evaluable patient. The Per-Protocol population (PP) (N = 12) included all patients of the ITT population but excluded patients with major protocol violations (0), the non-evaluable patients (1) and the wrongly included patients (0). The Safety Population included all patients enrolled in the study and who received at least one injection of TG1042 (N = 13). The efficacy analyses were done on ITT and PP populations. The ITT analysis was considered as the primary analysis of the study. All data analyses were performed using SAS® version 8.1 or higher. The best response rates were calculated with their exact 95% confidence interval using the Pearson-Clopper method. Duration of response was calculated for each patient with CR, PR or MR. The median time to progression was determined by Kaplan-Meier method. Its 95% confidence interval was calculated by the Brookmeyer-Crowley method.

## Results

### Study Population

**Figure 1:**
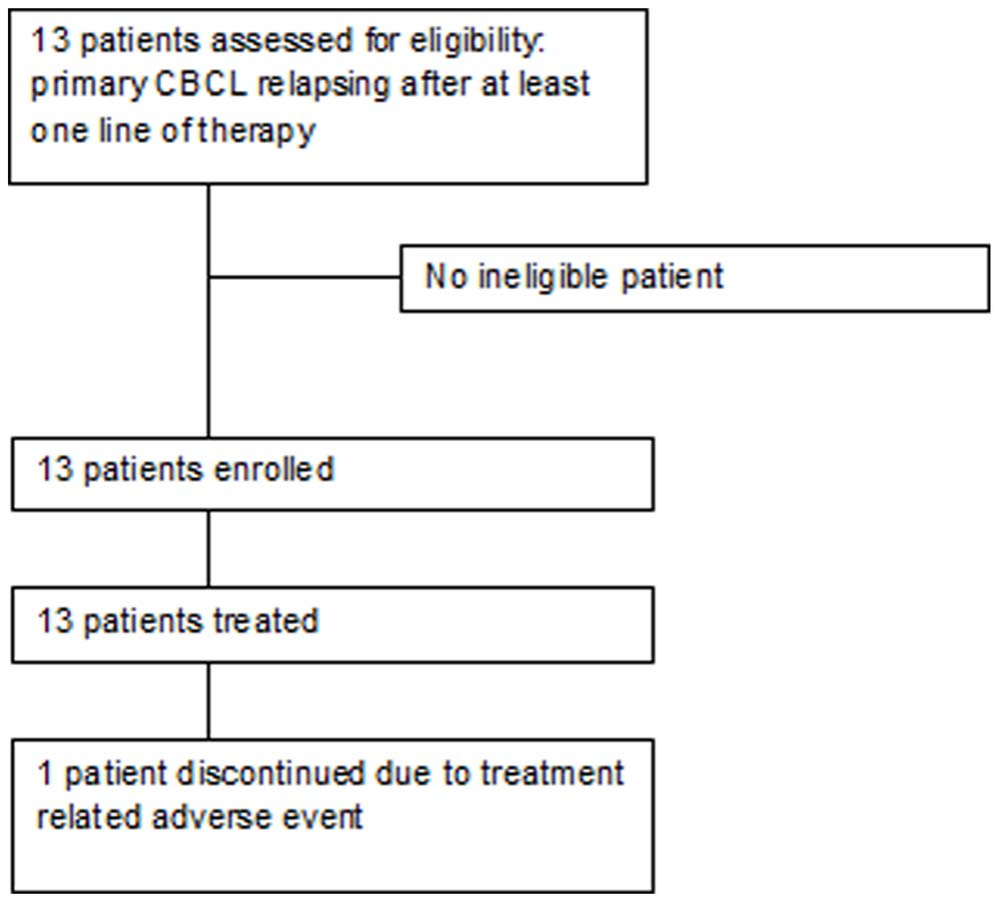
Study profile.

Thirteen patients were enrolled and treated by seven study centers between March 2007 and July 2008, last patient last visit was in January 2010 (Figure 1). Patient characteristics and previous treatments are shown in Table 1.

**Table 1:** Patient characteristics and clinical outcome.

Patient ID	Gender	Age	Disease Subtype	Previous Treatments	Local Response	Global Response	Duration of Response (days)	Time to Progression (days)
1	M	66	PCFCL	R, I	PR	PR	169	197
2	M	59	PCMZL	I	CR	CR	189	231
3	M	46	PCLBCL	R, C, I	CR	CR	1547	1602
4	F	40	PCMZL	R	CR	SD	274+	805+
5	M	58	PCFCL	S, R	PR	PR	191+	218+
6	F	40	PCMZL	R, C	NE	NE	NA	76+
7	F	39	PCFCL	I	CR	PR	1366+	1516+
8	M	30	PCMZL	R, C, I	PR	PR	631	813
9	F	50	PCMZL	R, C	PR	PR	624	707
10	M	58	PCFCL	I	CR	SD	554+	685+
11	M	80	PCFCL	R, I	CR	SD	1150+	1280+
12	M	57	PCMZL	R, C	SD	PD	NA	79+
13	F	70	PCFCL	R, I	CR	CR	239+	349

Response corresponds to the best response of the treated lesions observed during the study. Duration of response: Number of days between the documented best response (CR, PR, MR) of treated lesions until the first date of PD is objectively documented or the date of the last visit if no progression (censored data).

Time to progression: Number of days between the date of inclusion until the first date of PD of the treated lesions is objectively documented or the date of the last visit if no progression (censored data).

NA  =  Not applicable; NE  =  non evaluable; PR  =  partial response; SD  =  stable disease; CR  =  complete response.

PCFCL  =  Primary cutaneous follicle-center lymphoma, PCMZL  =  Primary cutaneous marginal zone lymphoma.

PCLBCL  =  Primary Cutaneous Diffuse Large B-cell Lymphoma, other than leg type

R  =  Radiotherapy, S  =  Surgery, C  =  Chemotherapy, I  =  Immunotherapy including interferon, imiquimod and rituximab.

“+”in seven patients means ongoing response or non-progression at the time of study database lock.

### Clinical Outcome

**Figure 2:**
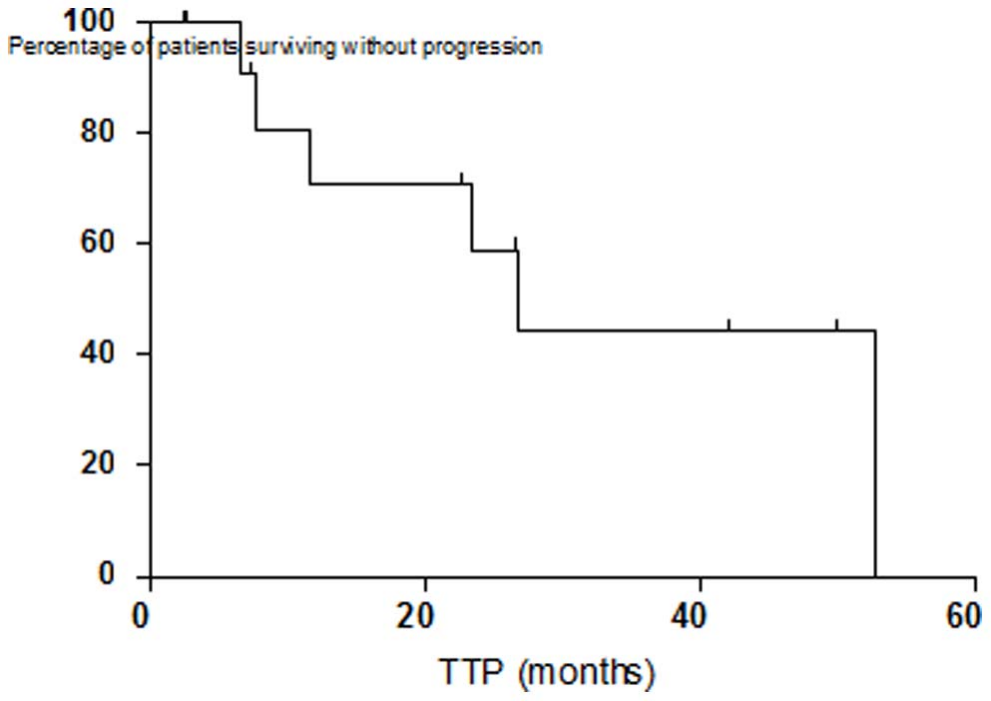
Time to Progression TTP).

The primary efficacy endpoint on study was the response rate of the treated lesions. Of the 13 patients treated with TG1042 intra-tumoral injections 12 were evaluable for response to treatment, all patients completed their follow-up as planned per-protocol. Eleven (85%, CI95%: 55–98) evaluable patients in the intent-to-treat population showed complete or partial response. Seven (54%) patients had a complete response and 4 (31%) patients had a partial response. One additional patient (8%) was considered as having a stable disease after the treatment. The secondary efficacy points of the study included duration of response for treated lesions, global response rate of treated and non-treated lesions and time-to-progression (summarized in Table 1). The median time to first objective response was 3.2 months (rage 1–17.5 months). In the seven patients who achieved a complete response the median time to complete response was 4.3 months (range 1.4–17.5). The median time to disease progression of the treated lesions was 23.5 months (range 6.5–26.4+ months) (Figure 2). Dermatology Life Quality Index (DLQI) showed a trend toward an improvement, the mean score was 2.1 at baseline (range 0–7) and decreased to 1.0 (range 0–3) after cycle 3. More precisely at baseline, the mean score of 7 patients who completed the DLQI was 2.1, indicative of a small effect on a patient's life. At the end of each cycle (Day 29) for up to 4 cycles, the mean score continued to decrease or remained stable: 2.0 at the end of cycle 1 (n = 9), 1.5 (cycle 2; n = 8), 1.0 (cycle 3; n = 8), and 1.2 (cycle 4; n = 5). The greatest mean change from baseline in the treatment period was -1.2, which was obtained at the end of cycle 3.

**Figure 3:**
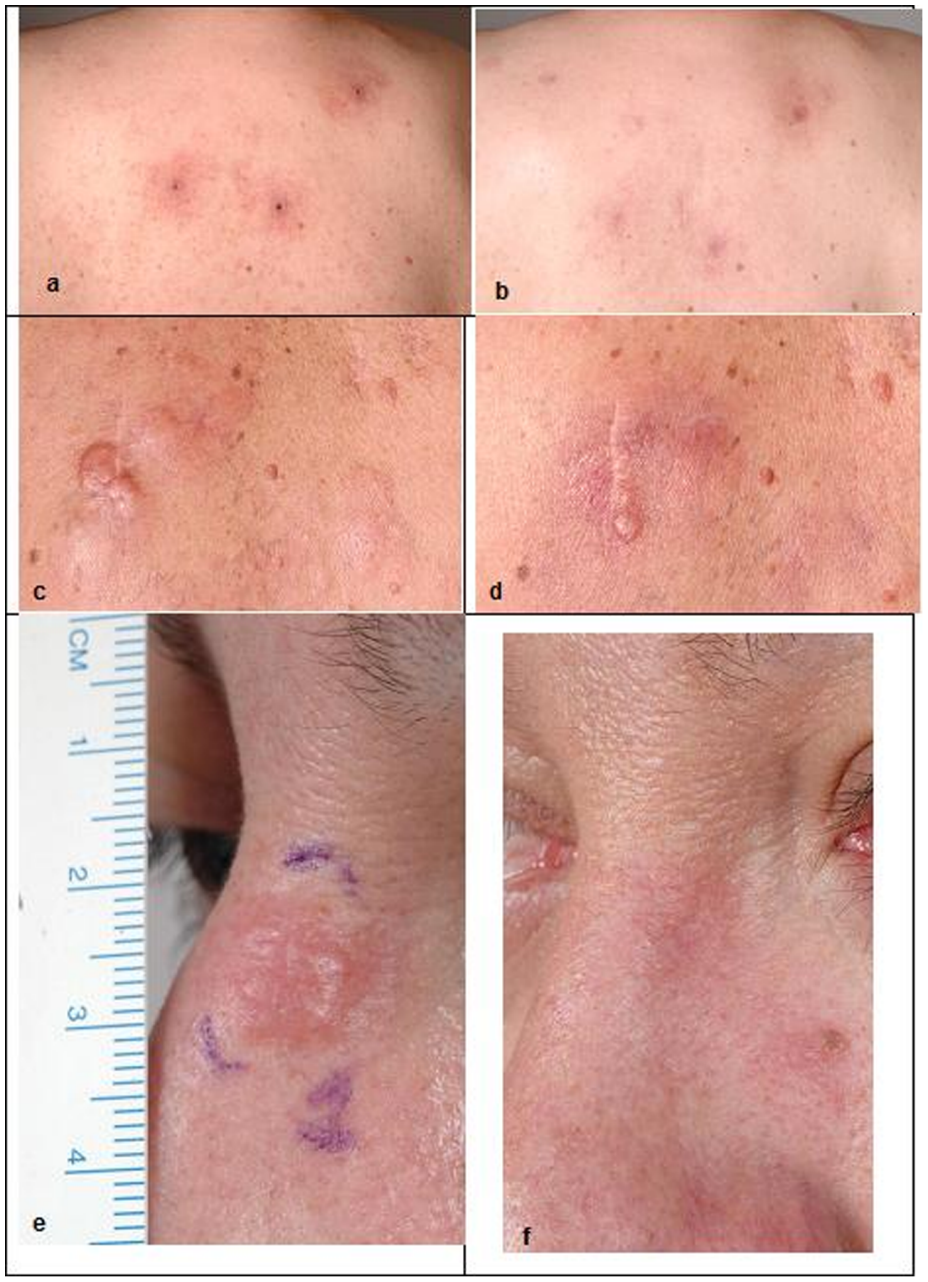
Examples of lesions before and after treatment with TG1042. Patient #2, PCMZL, before treatment (A) and after 7 months (B). Patient #5, PCFCL, before treatment (**C**) and after 4 months (**D**). Patient #7, PCFCL, before treatment (**E**) and after 9 months (**F**).

All reviewed skin biopsies showed an improvement of the lesions after treatment, characterized by an important decrease of the lymphoid infiltrate. Clinical examples of lesions before and after treatment are shown in Figure 3.

### Adverse Events

All 13 (100%) patients who received treatment with TG1042 were included in safety population. All patients experienced one or more Adverse Event (AEs) that was considered possibly or probably related to study drug (Table 2). One patient (No. 6) was discontinued from the study due to AEs of influenza-like illness, pyrexia, headache and skin blisters possibly related to TG1042.

**Table 2:** Adverse events reported.

Preferred Term	Number of patients	Number of events
Diarrhoea	3	13
Chills	4	11
Fatigue	8	17
Influenza like illness	4	17
Injection site erythema	4	10
Injection site irritation	5	6
Injection site pain	4	4
Pyrexia	6	17
Myalgia	3	18
Arthralgia	3	3
Headache	7	12

Only adverse events recorded in 3 patients or more have been listed in this table. A total of 196 AEs have been recorded, 179 of them were considered related to TG1042. All recorded adverse events were of grade 1 or 2 apart one grade 3 increase in blood lipase not considered related to TG1042.

With the exception of a single grade 3 event (increased lipase considered not related to TG1042 and which resolved without a treatment) all AEs were mild or moderate (grade 1 or 2) in severity. The most commonly reported AEs were fatigue (8/8 patients), headache (6/7 patients), pyrexia (6/6 patients), injection site irritation (5/5 patients), chills, influenza-like illness, injection site erythema and injection site pain (4/4 patients each), all of which were expected events associated with injections of TG1042. All reported adverse drug reactions resolved after treatment discontinuation. No clinically significant hematology or serum chemistry laboratory abnormalities were reported by the investigators.

## Discussion

This clinical study shows that repeated injections of TG1042 (adenovirus-IFNγ) into CBCL lesions lead to a reliable partial or total regression of the treated disease. This observation represents evidence that gene delivery based immunotherapy is feasible and can lead to sustained clinical tumor responses, which are of clinical benefit. The clinical improvement was supported by the improvement of the lesions seen at the pathological review of pre- and post-treatment biopsies. As expected, most commonly observed adverse events were injection site reactions and transient flu-like syndromes of mild or moderate intensity, demonstrating a good tolerability of the product.

The high response rate observed with TG1042 in this population of patients with CBCL raises the question of the mechanism of action of TG1042. It has been shown previously in patients treated with TG1042 for cutaneous lymphomas that not only the transgene IFNγ is expressed, but also a type I IFN response is induced due to the adenoviral vector itself. In addition, this type of combined type I (IFNα) and type II (IFNγ) IFN-response was predictive of the objective (partial and complete) clinical responses observed in the course of the treatment with TG1042 [12]. Similar activity can also be expected in CBCL, as it was described in other cancers such as melanoma [13], [14], [15], breast cancer [15], prostate cancer [16], renal cell carcinoma [17] and sarcoma [16] treated by gene therapy. A study performed in melanoma using the same adenoviral vector with interleukin-2 instead of interferon-γ showed regression of metastatic melanoma lesions in 6 out of 21 patients treated at the same dose of 3.10 [12] viral particles (vp) by injection [13].

This study has confirmed initial observation of the previous clinical trial, in which patients with CBCL already displayed a high response rate in general and as compared to what was observed in patients with CTCL. The high responsiveness of CBCL lesions to this approach in the absence of any cytotoxic treatment implies a high sensitivity of this tumor type to type I and II IFN-response. This argues in favor of immunologic approaches for the treatment of CBCL especially, since the treatment options for CBCL are rather limited and there are currently no registered drugs for this indication. The most commonly used approaches are still radiation therapy or surgery (see reference [7] for review), which show an initial response rate of up to 99%. However, due to the natural history of the disease up to 50% of the patients relapse in the decade following the initial treatment [18], .

The anti-CD20 monoclonal antibody, rituximab, offers also a new possibility of treatment in this type of lymphoma, it is more indicated in patients with multifocal lesions and the optimal treatment has not yet been well defined. In the literature, progression is observed in around 60% of cases with a median of 14.5 months. Intra lesional rituximab is an alternative to perfusions using lower doses than those used for intravenous therapy (less than 10%). The immediate response rate is high, close to 100% but is associated with a high rate of early recurrences, 42% in the year following treatment. The most frequently adverse events observed with rituximab are fever, chills, nausea, headache, urticaria and asthenia, which are mainly of grade 1 or 2. However one of the main concerns with rituximab, because of the long lasting complete depletion of B lymphocytes, is the risk of serious viral infections and especially progressive multifocal leukoencepahalopathy [20]. The side effects from the available treatments as well as their relapse rate leave the room for a treatment that would have good tolerability and could be easily repeated. From this standpoint the gene therapy approach with TG1042 evaluated in this study shows a favorable risk/benefit ratio with a high response rate and a long duration of response after treatment discontinuation. The adverse events are mild to moderate and easily manageable. TG1042 could therefore be an alternative to surgery or radiation therapy treatment or be used in combination with these therapies to minimize risk of recurrence; TG1042 could also be indicated in case of relapse. Further studies are necessary to evaluate the efficacy of TG1042 in comparison or in combination with standard treatment, such as radiotherapy.

## Supporting Information

Checklist S1 **TREND Statement Checklist for this manuscript.**
(PDF)

Protocol S1 **Clinical study protocol n°TG1042.06, final version, study phase II.**
(PDF)
