# Supplementary material for: TG1042 (Adenovirus-interferon-γ) in Primary Cutaneous B-cell Lymphomas: A Phase II Clinical Trial
Source: PLoS One. 2014 Feb 24;9(2):e83670. doi: 10.1371/journal.pone.0083670 (PMC3933342; doi:10.1371/journal.pone.0083670)
Supplement: Protocol S1 — Clinical study protocol n°TG1042.06, final version, study phase II. (PDF) [file pone.0083670.s002.pdf]

Clinical Study Protocol

CONFIDENTIAL

PHASE II CLINICAL TRIAL OF INTRA-LESIONAL ADMINISTRATION OF TG1042 (ADENOVIRUS-INTERFERON-γ) IN PATIENTS WITH RELAPSING PRIMARY CUTANEOUS B-CELL LYMPHOMAS.

PROJECT PROTOCOL N° TG1042.06
Final Version / 19 APR 2006

Study phase: II

SPONSOR: **Transgene S.A.**
11, rue de Molsheim
67082 STRASBOURG Cedex
FRANCE

PERSON TO CONTACT: **Pascal BLEUZEN, MD**
Clinical Project Manager
Medical and Regulatory Affairs Department
Phone: (33).(0)3.88.27.91.73
Fax : (33).(0)3.88.27.91.41

The confidential information contained in this document is the property of Transgene. It is provided to you in confidence, for review by you, your staff, regulatory authorities and members of ethics committees or institutional review boards. It is understood that this information will not be disclosed to any other third party, in any form, without prior authorization from Transgene, except to the extent necessary to obtain the written informed consent from the persons to whom the study drug may be administered.

DOCUMENT APPROVAL

SPONSOR'S OFFICERS

Pascal BLEUZEN, MD
Clinical Project Manager
Transgene

Signature Date

Marc BUYSE, ScD
Statistician
I.D.D.I.

Signature Date

SYNOPSIS

**Sponsor:** Transgene S.A.<br>**Study Drug**: TG1042 (ADENOVIRUS-INTERFERON-γ)	**Clinical Protocol:** TG 1042.06
**Study** PHASE II CLINICAL TRIAL OF INTRA-LESIONAL ADMINISTRATION OF TG1042 (ADENOVIRUS-INTERFERON-γ) IN PATIENTS WITH RELAPSING PRIMARY CUTANEOUS B-CELL LYMPHOMAS.	
**Study Period:** Q2 2006 – Q2 2008	**Clinical Phase:** II

Objectives:
The objective of this study is to evaluate the efficacy (primary objective) and safety (secondary objective) of a four-month dosing period of intra-lesional injection of TG1042 in patients with relapsing primary cutaneous B-cell lymphoma (CBCL).

Methodology:
This study is a phase II, multicenter, study of repeated intra-lesional administration of TG1042 in patients with relapsing CBCL.

Number of Patients:
Fourty one patients (13 in the 1st step, 28 in the 2nd step) in approximately 15 centers are planned to be included in the study.

Diagnosis and Inclusion Criteria:
Written informed consent from patient. Primary CBCL (excluding diffuse large B-cell, leg type and intravascular large B-cell lymphoma). Histology consistent with primary CBCL. No extracutaneous involvment. ECOG performance status of 0, 1. Relapse or active disease after radiotherapy or other standard therapy if radiotherapy is contra-indicated. Minimum life expectancy > 3 months. Adequate blood count. Adequate hepatic and renal function. Age ≥ 18 years and <80 years old. No prior treatment for CBCL within the preceding 4 weeks.

Test Product, Dose, Route of Administration:
Intra-lesional TG1042 at the dose of $5x10^{10}$ viral particles (vp) per lesion will be administered into up to six lesions at days 1, 8, 15 of a monthly cycle.

Reference Therapy, Dose, Route of Administration:
Not applicable

Duration of Treatment:
Patients will be treated for up to 4 cycles (12 injections) i.e. 16 weeks

Criteria for Evaluation:
Primary efficacy endpoint: response rate.
Secondary efficacy endpoints: duration of response, time to progression, dermatology life quality index.
Secondary safety endpoints: incidence of adverse events, incidence of serious adverse events.

Statistical Methods:
The following common assumptions are made:

- The inactivity cut-off is chosen equal to 50%, the activity cut-off equal to 75%. Hence the hypotheses of interest are H_0: r ≤ 50% against H_A: r ≥ 75%, where r is the response rate
- The type I error rate (α, probability of accepting an insufficiently active treatment, a false positive outcome) is set to 5%
- The type II error rate (β, probability of rejecting an active treatment, a false negative outcome) is set to 10%

Under these assumptions, an optimal design consists of the following two stages:

1. treat 13 evaluable patients
   - if at most 7 responses are observed, stop the trial and declare the drug insufficiently active
   - otherwise continue patient entry
2. treat additional evaluable patients up to a total of 41
   - if at most 25 responses are observed, declare the drug insufficiently active
   - if at least 26 responses are observed, declare the drug active

With this design, if the response rate is truly equal to 50%, the expected sample size is 27 evaluable patients and the maximum sample size is 41 evaluable patients.
Response rates and incidence will be compared by a Chi-squared test. Time to progression, duration of response will be estimated by the Kaplan-Meier methods.

TABLE OF CONTENTS

1 RATIONALE 9
1.1 DESCRIPTION OF CUTANEOUS B-CELL LYMPHOMA 9
1.2 STUDY DRUG RATIONALE 12
1.3 AD-IFNγ IN CBCL 15
2 OBJECTIVES 17
2.1 PRIMARY OBJECTIVE 17
2.2 SECONDARY OBJECTIVE 17
3 STUDY DESIGN 18
3.1 OVERALL STUDY DESIGN AND PLAN DESCRIPTION 18
3.2 DISCUSSION OF THE STUDY DESIGN 18
3.3 NUMBER OF CENTERS AND PATIENTS 18
3.4 PATIENT ACCRUAL AND DURATION OF STUDY 18
4 STUDY POPULATION 18
4.1 INCLUSION CRITERIA 18
4.2 EXCLUSION CRITERIA 19
4.3 THERAPY RESTRICTIONS 19
5 STUDY DRUG 20
5.1 CHARACTERISTICS AND SUPPLY 20
5.2 PACKAGING AND LABELING 20
5.3 CONDITIONS OF DRUG STORAGE AND USE 20
5.4 PREPARATION FOR ADMINISTRATION 20
5.5 WASTE HANDLING PRECAUTIONS 20
6 TREATMENT PLAN 21
6.1 TREATMENT ADMINISTERED 21
6.2 METHOD OF ASSIGNING PATIENTS TO THE TREATMENT GROUP 22
6.3 SAFETY STOPPING RULES 23
6.4 BLINDING/UNBLINDING 23
6.5 PREMATURE WITHDRAWAL OF PATIENTS 23
6.6 REPLACEMENT POLICY 23
7 STUDY VISITS AND PROCEDURES 24
7.1 ASSESSMENTS TO BE PERFORMED 24
7.2 BASELINE: ON DAYS -14 TO 0 24
7.3 WHILE ENROLLED ON THE PROTOCOL 25
7.4 END-OF-TREATMENT VISIT (TO BE CONDUCTED 4 WEEKS AFTER DAY 29 OF THE LAST CYCLE): 25
7.5 POST-TREATMENT FOLLOW-UP 25
8 TUMOR EVALUATION 26
8.1 BASELINE EVALUATION 26
8.2 ON TREATMENT EVALUATION 27
8.3 RESPONSE CRITERIA 28
8.4 TUMOR IMAGING 28
9 ASSESSMENT OF SAFETY 28
9.1 DEFINITIONS 28
9.2 INTENSITY, RELATIONSHIP AND OUTCOME EVALUATION 30
9.3 PRE-DRUG EVENT MANAGEMENT 31
9.4 ADVERSE EVENT MANAGEMENT 32
9.5 SERIOUS ADVERSE EVENT MANAGEMENT 33
9.6 LABORATORY VALUES, VITAL SIGNS, PHYSICAL FINDINGS AND OTHER SAFETY DATA 35

10 STATISTICAL METHODS PLANNED AND SAMPLES SIZE 35
10.1 DETERMINATION OF SAMPLE SIZE 35
10.2 STUDY ENDPOINTS 35
10.3 DISPOSITION OF PATIENTS 36
10.4 PROTOCOL DEVIATIONS 36
10.5 STATISTICAL AND ANALYTICAL PLANS 36
10.6 DATA SETS ANALYZED 37

11 CHANGES IN THE CONDUCT OF THE STUDY 38
11.1 PROTOCOL AMENDMENTS 38
11.2 PREMATURE STUDY TERMINATION 38

12 ETHICAL CONSIDERATIONS 38
12.1 INDEPENDENT ETHICS COMMITTEE/INSTITUTIONAL REVIEW BOARD 38
12.2 INFORMED CONSENT 38
12.3 CONFIDENTIALITY OF PATIENT DATA 39
12.4 DATA SAFETY MONITORING COMMITTEE 39

13 REGULATORY CONSIDERATIONS 41
13.1 REGULATORY CONSIDERATIONS 41
13.2 REGULATORY APPROVAL / AUTHORIZATION 41
13.3 INVESTIGATORS OBLIGATIONS 41
13.4 INSURANCE 42

14 QUALITY CONTROL AND QUALITY ASSURANCE 42
14.1 SOURCE DATA AND DOCUMENTS 42
14.2 PERIODIC MONITORING 42
14.3 AUDIT AND INSPECTION 43

15 DATA HANDLING AND RECORD KEEPING 43
15.1 INVESTIGATORS INFORMATION 43
15.2 CASE REPORT FORMS (CRF) 43
15.3 CHANGES TO CRF DATA 43
15.4 PROVISION OF ADDITIONAL INFORMATION 44

16 REPORTING AND PUBLICATION 44
16.1 CLINICAL STUDY REPORT 44
16.2 CONFIDENTIALITY OF STUDY DATA 44
16.3 PUBLICATION POLICY 44

17 ARCHIVING 45
17.1 INVESTIGATOR SITE FILE 45
17.2 TRIAL MASTER FILE 45

18 REFERENCES 46

19 APPENDICES 47
APPENDIX A: FLOW-CHART OF VISITS AND PROCEDURES 48
APPENDIX B: DERMATOLOGY LIFE QUALITY INDEX[9] 49
APPENDIX C: PHOTOGRAPHIC PROCEDURE 50
APPENDIX D: INCLUSION PROCEDURE 53
APPENDIX E: PERFORMANCE STATUS (ECOG SCALE) 54

ABBREVIATIONS AND DEFINITION OF TERMS

ABBREVIATIONS	MEANING OF ABBREVIATIONS IN DOCUMENT
AE	Adverse Event
Ad	Adenovirus
Ad-IFNγ	Adenovirus-Interferonγ
ALT	Alanine Amino-Transferase
AST	Aspartate Amino-Transferase
CA	Composite Assessment
CBCL	Cutaneous B-Cell Lymphomas
CD	Cluster of differentiation
CR	Complete Response
CRF	Case Report Form
CTCAE	Common Toxicity Criteria for Adverse Event
CTCL	Cutaneous T-Cell Lymphoma
CPM	Clinical Project Manager
DLQI	Dermatology Life Quality Index
DLT	Dose Limiting Toxicity
DNA	Deoxyribonucleic Acid
DSMC	Data Safety Monitoring Committee
ECG	Electrocardiogram
ECOG	Eastern Cooperative Oncology Group
EORTC	European Organization for Research and Treatment of Cancer
GCP	Good Clinical Practice
ICH	International Conference on Harmonization
IEC	Independent Ethics Committee
IFN	Interferon
Ig	Immunoglobulin
IL	Interleukin
IP10	Interferon-inducible protein 10
IRB	Institutional Review Board
LN	Lymph Node
NCR	No Carbon Required
MALT	Mucosa-Associated Lymphoid Tissue Lymphoma
MHC	Major Histocompatibility Complex
mRNA	Messenger Ribonucleic Acid
MTD	Maximal Tolerated Dose
NCI	National Cancer Institute
PC	Personal Computer
PCFCL	Primary Cutaneous Follicle Center Lymphoma
PCLBCL	Primary Cutaneous Diffuse Large B-Cell Lymphoma
PCMZL	Primary Cutaneous Marginal Zone B-Cell Lymphoma
PD	Progression Disease
PDE	Pre-Drug Event

ABBREVIATIONS	MEANING OF ABBREVIATIONS IN DOCUMENT
PET	Positron-Emission Tomography
PR	Partial Response
rIFN	Recombinant Interferon
SAE	Serious Adverse Event
SD	Stable Disease
SP	Sum of Products
Th	T helper
TBI	Tumor Burden Index
TNF	Tumor necrosis factor
TNM	Tumor Node Metastase (Tumor classification)
vp	Viral Particle
WBC	White Blood (cell) Count
WHO	World Health Organization

DEFINITION OF TERMS

Non evaluable patient
A non evaluable patient will be a patient for whom, for any reason, no efficacy evaluation by local evaluator has been undertaken. A patient for whom any efficacy evaluation will be missing will be considered as evaluable if at least one evaluation is available. Non evaluable patient will be included in the "intention-to-treat" analysis as failure (i.e. progression disease) but not in the "per-protocol" analysis.

Wrongly included patient
Patients who will be included but who will meet one of the following criteria will be defined as "wrongly included" patient:

- No histological documentation of cutaneous B-cell lymphoma (CBCL).
- No prior radiotherapy or no other standard therapy if radiotherapy is contra-indicated.

Wrongly included patient will be included in the "intention to treat" analysis of efficacy and in the safety analysis but not in the "per protocol" analysis of efficacy.

Pre-selected patient
Every patient examined by the investigator during the inclusion period and who will satisfy the following criteria will be defined as "pre-selected patient":

- History of CBCL (whatever the subtype).
- Relapse after radiotherapy or other standard therapy if radiotherapy is contra-indicated.

PHASE II CLINICAL TRIAL OF INTRA-LESIONAL ADMINISTRATION OF TG1042 (ADENOVIRUS-INTERFERON-γ) IN PATIENTS WITH RELAPSING PRIMARY CUTANEOUS B-CELL LYMPHOMAS.

1 RATIONALE

1.1 Description of Cutaneous B-Cell Lymphoma

Primary cutaneous B-Cell Lymphomas (CBCL) are characterized by a clonal accumulation of malignant lymphocytes in the skin. In a strict sense, they are extra-nodal Non-Hodgkin Lymphomas primarily manifesting in the skin and being confined to the skin. The skin is the second most common site of extranodal Non-Hodgkin Lymphomas after the stomach. The distinction between CBCL and primary nodal lymphomas of the same histologic subtype, involving the skin secondarily is essential, since lymphomas with identical cytomorphology may benefit of different therapeutic approaches and may have completely different outcomes if derived from extra-cutaneous sites. This special behavior of primary CBCL justifies special attention and requires to exclude extra-cutaneous involvement before starting any treatment.

The WHO/EORTC 2005 classification[1] which describes 5 main different subtypes of primary CBCL:

- Primary cutaneous marginal-zone B-cell lymphoma (PCMZL);
- Primary cutaneous follicle-center lymphoma (PCFCL);
- Primary cutaneous diffuse large B-cell lymphoma (PCLBCL), leg type;
- Primary cutaneous diffuse large B-cell lymphoma, other;
- Intravascular large B-cell lymphoma.

This study plans to include the PCMZL, PCFCL and primary cutaneous diffuse large B-cell lymphoma, other subtypes.

1.1.1 Primary cutaneous marginal-zone B-cell lymphoma

PCMZL is an indolent lymphoma composed of small B cells, including marginal zone (centrocyte-like) cells, lymphoplasmacytoid cells and plasma cells. PCMZL is considered part of the broad group of extranodal marginal zone B-cell lymphoma commonly involving mucosal sites, called MALT (mucosa-associated lymphoid tissue) lymphoma.
In most instances patients with PCMZL present with red to violaceous papules, plaques, or nodules localized preferentially on the trunk or extremities, especially the arms. In contrast to follicle center lymphoma, presentation with multifocal skin lesions is frequent. Ulceration is uncommon. PCMZLs have a tendency to recur in the skin, but dissemination to extracutaneous sites is exceedingly rare. In some cases spontaneous resolution of the skin lesions may be observed. An association with Borrelia burgdorferi infection has been reported in a significant minority of European cases of PCMZL, but not in Asian cases or cases from the United States.

These lymphomas show nodular to diffuse infiltrates with sparing of the epidermis. The infiltrates are composed of small lymphocytes, marginal zone B cells (centrocyte-like cells), lymphoplasmacytoid cells and plasma cells, admixed with small numbers of centroblast- or immunoblast-like cells and many reactive T cells. Reactive germinal centers are frequently observed. The marginal zone B cells express CD20, CD79a and bcl-2, but are negative for CD5, CD10 and bcl-6, which may be useful in distinction from PCFCL. Reactive germinal centers are typically bcl-6^{+}, CD10^{+} and bcl-2^{-}. Plasma cells express CD138 and CD79a, but generally not CD20 and show monotypic cytoplasmic immunoglobulin (Ig) light chain expression on paraffin-embedded sections.
The prognosis of PCMZL is excellent with a 5-year survival close to 100%.
Patients with a solitary or a few lesions can be treated with radiotherapy or surgical excision. In patients with associated B burgdorferi infection, systemic antibiotics should be tried first. For patients presenting multifocal skin lesions, chlorambucil or intralesional or subcutaneous administration of interferon alpha may produce complete responses in approximately 50% of patients. Very good results have also been obtained with the use of systemic or intralesional anti-CD20 antibody (rituximab). In patients showing frequent skin relapses, topical or intralesional steroids may be considered; alternatively, an expectant strategy can be followed, similar to that used in other indolent B-cell lymphomas and leukemias.

1.1.2 Primary cutaneous follicle-center lymphoma

PCFCL is defined as a tumor of neoplastic follicle center cells, usually a mixture of centrocytes (small and large cleaved follicle center cells) and variable numbers of centroblasts, with a follicular, a follicular and diffuse, or a diffuse growth pattern, which is generally present on the head or trunk.
PCFCL has a characteristic clinical presentation with solitary or grouped plaques and tumors, preferentially located on the scalp or forehead or on the trunk and rarely on the legs. Particularly on the trunk, these tumors may be surrounded by erythematous papules and slightly indurated plaques, which may precede the development of tumorous lesions for months or even many years. Presentation with multifocal skin lesions is observed in a small minority of patients, but is not associated with a more unfavorable prognosis. If left untreated, the skin lesions gradually increase in size over years, but dissemination to extracutaneous sites is uncommon.
PCFCLs show nodular to diffuse infiltrates with almost constant sparing of the epidermis. The histologic picture is variable, which relates primarily to the age and the growth rate of the biopsied skin lesion as well as the location. A clear-cut follicular growth pattern is more commonly observed in lesions arising on the scalp than those presenting on the trunk. Small and early lesions contain a mixture of centrocytes, relatively few centroblasts and many reactive T cells. Large centrocytes, often multilobated, are a common feature of PCFCL. The neoplastic cells express the B-cell–associated antigens CD20 and CD79a and may show monotypic staining for surface immunoglobulins. Staining for CD5 and CD43 is negative.
Clonally rearranged Ig genes are present. Somatic hypermutation of variable heavy and light chain genes has been demonstrated, which further supports the follicle center cell origin of these lymphomas. Inactivation of p15 and p16 tumor suppressor genes by promotor hypermethylation has been reported in about 10% and 30% of PCFCLs, respectively. Chromosomal imbalances have been identified by comparative genomic hybridization analysis in a minority of PCFCLs,

but a consistent pattern has not yet emerged. PCFCLs have the gene expression profile of germinal center–like large B-cell lymphomas.
In patients with localized or few scattered skin lesions, radiotherapy is the preferred mode of treatment. Recent studies report beneficial effects of systemic or intralesional administration of anti-CD20 antibody (rituximab) therapy in small series of PCFCLs, but the long-term effects of this approach have still to be determined.

1.1.3 Primary cutaneous diffuse large B-cell lymphoma, leg type

PCLBCL, leg type, is a PCLBCL with a predominance or confluent sheets of centroblasts and immunoblasts, characteristically presenting with skin lesions on the (lower) legs. Uncommonly, skin lesions with a similar morphology and phenotype can arise at sites other than the legs.
PCLBCL, leg type, predominantly affects elderly patients, particularly females. Patients present with generally rapidly growing red or bluish-red tumors on one or both (lower) legs. In contrast to the group of PCFCLs, these lymphomas more often disseminate to extracutaneous sites and have a more unfavorable prognosis.
These lymphomas show diffuse infiltrates, which often extend into the subcutaneous tissue. These infiltrates generally show a monotonous population or confluent sheets of centroblasts and immunoblasts. Mitotic figures are frequently observed. Small B cells are lacking and reactive T cells are relatively few and often confined to perivascular areas. A prominent stromal reaction as in PCFCLs is not observed.
The neoplastic B cells express antigens CD20 and CD79a. In contrast to the group of PCFCLs, PCLBCL, leg type, shows strong bcl-2 expression, also in cases not located on the legs. Bcl-6 is expressed in most cases, whereas CD10 staining is generally absent.
The t(14;18) is not found in PCLBCLs, although strong bcl-2 expression is common in this group. In some cases bcl-2 overexpression may result from chromosomal amplification of the bcl-2 gene. Inactivation of p15 and p16 tumor suppressor genes by promotor hypermethylation has been detected in 11% and 44% of PCLBCLs, respectively. Chromosomal imbalances have been identified in up to 85% of PCLBCLs, with gains in 18q and 7p and loss of 6q as most common findings. Recent studies demonstrated translocations involving myc, bcl-6 and IgH genes in 11 of 14 PCLBCL-leg cases, but not in patients with a PCFCL with a diffuse infiltration of large centrocytes. Recent studies suggest that patients with PCLBCL-leg have an activated B-cell gene expression profile.
The 5-year survival of 78 cases included in the Dutch and Austrian registries was 55%. PCLBCLs on the leg have an inferior prognosis compared to PCLBCLs presenting at other sites. The presence of multiple skin lesions at diagnosis is a significant adverse risk factor. In a recent study, patients presenting with a single skin tumor on one leg had a disease-related 5-year survival of 100%, whereas patients presenting with multiple skin lesions on one or both legs had a disease-related 5-year survival of 45% and 36%, respectively.
These lymphomas should be treated as systemic diffuse large B-cell lymphomas with anthracycline-based chemotherapy. In patients presenting with a single small skin tumor, radiotherapy may sometimes be considered. Systemic administration of anti-CD20 antibody (rituximab) has been proved to be effective in some patients, but long-term follow-up data are not available and the place of rituximab in the treatment of PCLBCL, either as single agent therapy or in combination with systemic chemotherapy remains to be established.

1.1.4 Primary cutaneous diffuse large B-cell lymphoma, other

The term "PCLBCL, other," refers to rare cases of large B-cell lymphomas arising in the skin, which do not belong to the group of PCLBCL, leg type, or the group of PCFCLs. These cases include morphologic variants of diffuse large B-cell lymphoma, such as anaplastic or plasmablastic subtypes or T-cell/histiocyte rich large B-cell lymphomas. Such cases are generally a skin manifestation of a systemic lymphoma.

1.1.5 Intravascular large B-cell lymphoma

Intravascular large B-cell lymphoma is a well-defined subtype of large B-cell lymphoma, defined by an accumulation of large neoplastic B cells within blood vessels. These lymphomas preferentially affect the central nervous system, lungs and skin and are generally associated with a poor prognosis.
Patients often have widely disseminated disease, but cases with only skin involvement may occur. Clinically, intravascular large B-cell lymphoma may present with violaceous patches and plaques or teleangiectatic skin lesions usually on the (lower) legs or the trunk. Patients presenting with only skin lesions appear to have a significantly better survival than patients with other clinical presentations (3-year overall survival: 56% versus 22%).
Multiagent chemotherapy is the preferred mode of treatment, also in patients presenting with skin-limited disease.

1.2 Study drug rationale

1.2.1 Interferon γ (IFNγ)

1.2.1.1 Antitumor activity of IFNγ

The antitumor activity[a] of IFNγ is a result of the antiproliferative, immunostimulatory and gene activity modulating effects of this cytokine. Evidently in different types of malignancy, one or another activity may be dominant.

1.2.1.1.1 Antiproliferative activity of IFNγ

IFNγ suppresses the proliferation of various normal and malignant cells. In most cases, especially in malignant cells, the antiproliferative activity of IFNγ is accompanied by a differentiating effect. In some cell types the antiproliferative activity may lead to apoptosis.
The direct antiproliferative effect of IFNγ may be due to several different mechanisms.

- The most widely accepted and discussed mechanism is the suppression of oncogene expression and more specifically the proto-oncogene c-myc.
- Induction of the enzyme indolamine-2, 3-dioxygenase leading to tryptophan starvation.
- Decreased expression of transferrin receptors leading to iron deficiency.

---

[a] For an in depth review of antitumor activity of IFNγ see [10]

- Induction of certain proteins such as death associated proteins and calcium/calmodulin-dependent enzymes leading to apoptosis.
- Induction of Fas, leading to apoptosis.
- Blockade of cell cycle at the end of the G1 phase by inducing the synthesis of the kinase inhibitor $p27^{kip1}$.
- Inhibition of growth factor production.
- Inhibition of deoxyribonucleic acid (DNA) synthesis and more specifically of the DNA polymerase.
- Stimulation of an angiostatic activity.

Most probably the direct antiproliferative activity of IFNγ is due to a combination of some of these mechanisms and some others may yet be discovered.

1.2.1.1.2 Immunostimulatory activity of IFNγ

Stimulation of cellular immunity is one of the main biological functions of IFNγ. It is manifested by activation of effector cells of the cellular immunity (macrophages, natural killer /CD56 cells, CD8+ cytotoxic T lymphocytes, CD4+ T helper (Th) cells) which destroy foreign pathogens and tumor cells.
The major cells of the immune system activated by IFNγ are the macrophages. IFNγ regulates the differentiation of monocytes into mononuclear phagocytes (macrophages), stimulates the antigen-presenting activity of macrophages by the regulation of the expression of major antigens of the Major Histocompatibility Complex (MHC) and facilitates their interaction with T cells.
IFNγ also participates in the regulation of Th cell differentiation by stimulating the proliferation of Th1 and inhibiting the proliferation of the antagonistic Th2 type favoring a cellular instead of a humoral immune response. IFNγ exhibits a stimulatory effect not only on CD4+ but also on CD8+ lymphocytes. It is believed that IFNγ also participates in switching the Ig isotype in B lymphocytes.

1.2.1.1.3 Modulation of gene activity by IFNγ

IFNγ suppresses the activity of some genes, such as, for example, some oncogenes, genes coding for collagen, for transferrin receptors and activates others like the genes of interleukin (IL)2, IL12, tumor necrosis factor (TNF)-α, of IL2 receptors, of MHC I and MHC II.
It is believed that in most cells IFNγ activates 15 to 20 genes, which usually occurs at the level of transcription. There are data showing that the mechanisms of gene activation by IFNγ are different from those of IFNα.
IFNγ also induces the intercellular adhesion molecule I, which plays an important role in the adhesion of loose cancer cells and the formation of metastases. On the other hand, due to the intercellular adhesion molecule I, tumoricidal lymphocytes can attach to cancer cells and destroy them.
It should be stressed that the effect of IFNγ on gene activity is not precisely defined and depends on a number of factors such as, for example, cell type, animal species and body regions from which the cells have been isolated.

1.2.1.2 IFNγ in clinical trials for solid tumors

Based on the properties of IFNγ described above, the use of recombinant IFNγ (rIFNγ) protein as a potential anticancer therapeutic has been widely investigated in clinical trials. An objective response rate between 5 and 10% has been obtained in patients with renal cell carcinoma and melanoma[2-4]. Notably, the overall effectiveness of treatment depended on the dose given, schedule of treatment, route of administration and type of cancer. Higher doses of IFNγ resulted generally in better response rates. However, as often observed with cytokines given by the systemic route, severe systemic toxicities with high-dose IFNγ therapy limited the effectiveness of this approach.
A phase II/III randomized, controlled clinical study conducted in 148 patients with ovarian cancer showed that subcutaneous injections of rIFNγ, combined with chemotherapy, yielded a benefit in prolonging progression-free survival[5].

1.2.2 The Parental Adenovirus

Adenovirus[b] (Ad) can be isolated from numerous animal species in which they cause pharyngitis, enteritis, pneumonia and keratitis. Among the currently known serotypes, 34 have been isolated from humans. Some of them (serotypes 3, 4, 7, 14) are responsible for acute respiratory infections, against which a multivalent vaccine was developed. Other serotypes (1, 2, 5, 6, etc.) remain latent in lymphoid tissues and can be isolated from the in vitro culture of these tissues. Finally, some serotypes (12, 18, etc.) are not associated with any of the above mentioned properties[6]. Human Ads can be classified according to their tumorigenicity in newborn hamsters. They are classically divided into strongly oncogenic, weakly oncogenic and non-oncogenic. All Ads, however, are capable of transforming rat embryonic cells in vitro.
The Ad consists of a DNA molecule associated with proteins surrounded by an icosahedral capsid of 60-90 nm in diameter. The genome of the type 5 Ad is a double-stranded DNA molecule of 36 kbp coding for at least 30 proteins. An inverted terminal repetition of 103 bp is present at the extremities of the molecule. The viral particles (vp) adhere to the cell membrane then penetrate into the cell via specific Ad receptors. After uncoating, the viral DNA with associated proteins migrates into the nucleus, where its transcription by the host enzymes is initiated. The replication cycle is conventionally divided into two phases of expression of the viral genome. These two phases, early and late, are separated by the period of viral replication, 5 to 6 hours after infection. A complete cycle lasts about 30 hours.
The parental virus used for the construction of TG1042 is the human Ad type 5 which was deleted of its E1 and E3 regions. The vector cannot replicate autonomously, due to this deletion of an essential region of its genome.
The E1A gene is transcribed immediately after infection. It codes for the proteins that control the transcription of other early genes. It also makes the host cells sensitive to TNF, thereby favoring their destruction. However, if cell death occurs too rapidly, it deprives the virus of the cellular mechanism necessary to ensure its replication. This is why another gene, E1B, restrains the expression of the early genes, slowing down the destruction of the host cell. It also furthers the expression of the late viral genes. The proteins necessary for the replication of the viral genome are coded by the E2 region. The E3 region codes for the proteins that allow the infected cells to

[b] Further information about the study drug is available in the Investigator's Brochure.

escape from the immune system. The E4 region is essential for the multiplication of DNA and favors the expression of the late genes. Among the eight proteins coded by this region, one controls the transcription of the E2 region and participates in the replication of the viral DNA, another is necessary for the expression of the late genes and the arrest of the functions of the host cell.
During the late phase, the synthesis of host cell proteins is blocked and the transcription of the late viral genes leads to the synthesis of structural viral proteins allowing new viral particles to be assembled. During an infection cycle, one Ad can produce up to 10,000 daughter particles.
Further information about the adenoviral vector is available in the Investigator's Brochure.

1.2.3 Rationale of Ad-IFNγ approach

As it is often observed with cytokines given systemically, severe systemic toxicities with high-dose IFNγ therapy have limited the effectiveness of this therapy. To overcome the issues associated with systemic toxicity, the direct intra-lesional administration of rIFNγ has been attempted in solid tumors, but the short half life of rIFNγ has limited the interest in this approach.
Based on the properties of IFNγ described above, the rationale for using Ad-IFNγ consists of delivery directly into the tumor micro-environment with the adenoviral vector. Transcription and translation of the IFNγ DNA in the infected cells will lead to sustained local concentration of IFNγ protein without the need of infecting all the cells present in the tumor mass and keeping systemic IFNγ levels low enough to prevent toxic effects.
A similar approach was initially conducted with a retroviral vector expressing IFNγ. Encouraging results were obtained after single or multiple direct intra-lesional injections of this vector in patients with metastatic melanoma.
In preclinical experiments, the antitumoral effect of Ad-IFNγ has been assessed in animal models. Direct delivery of Ad-IFNγ into established B16F0 tumors in mice induces a decrease of tumor growth leading to a significant increase in survival rate which could be explained by a significant intra-lesional expression of IFNγ. Encouraging results were also obtained in the RENCA pulmonary metastases murine model where a 90 % inhibition in the number of metastatic pulmonary nodules was observed in animals receiving Ad-IFNγ, resulting in a significant increase in survival as compared to the control groups.

1.3 Ad-IFNγ in CBCL

1.3.1 Immune biology of CBCL with respect to IFNγ

1.3.1.1 Nodal B-cell lymphoma and Th1/Th2 switch

The clinical course of B-cell lymphoma is accompanied by a dysregulated synthesis of cytokines. Cytokines can influence tumor cells in an autocrine, paracrine and endocrine fashion. Regarding the preferentially produced cytokines, two major subdivisions of the Th system can be defined: Th1 clones secrete mainly IL2 and IFNγ, whereas Th2 clones produce IL4, IL5, IL6 and IL10. Th1 cells are involved in cell-mediated immune response. Th2 cells encourage antibody production, in particular IgE responses and enhance eosinophil proliferation and function. Since Th1 cells are the principle effectors for cell-mediated immunity against tumor cells and delayed-

type hypersensitivity reaction, it seems to be an advantage for the malignant cells to switch the immune response of the host to a Th2 type.
Numerous findings suggest that a Th1/Th2 imbalance could play a central role in B-cell lymphoma:

- IL10 is clearly overexpressed in B-cell lymphoma.
- IL-10 is the most relevant cytokine for B-cell survival in mice and humans.
- IL-10 is a critical factor for the progression of the B-cell malignant disease.
- The transcriptional repressor protein BCL-6, implicated in the pathogenesis of B cell lymphoma, is a major negative regulator of Th2 differentiation and Th2-type inflammation.
- IL-4 and IL-5 can serve as growth factors for malignant B cells and enhance tumor growth.
- IL10 indirectly prevents antigen-specific T cell activation, which is in turn associated with down-regulation of antigen presentation and accessory cell functions of monocytes, macrophages, Langerhans cells and dendritic cells. In addition, IL10 limits T cell expansion by directly inhibiting IL2 production by these cells.

1.3.1.2 Cutaneous B-Cell Lymphoma

The data on physiopathogy of CBCL are limited. Physiologically, B-lymphocytes are not present in the skin. Even in pathological situations, they rarely occur. A Th2 pattern is also found in CBCL lesions which might contribute to tumor B-cell growth. Indeed, an overexpression of TNFα, IL10 and IL6 is found in CBCL lesions.

1.3.2 Previous Clinical Results with Ad-IFNγ: the phase I/II study.

A clinical trial study entitled: Phase I/II clinical trial of biologic therapy with intratumoral TG1042 (Adenovirus-Interferon-γ) in patients with advanced cutaneous T-cell lymphomas (CTCL) - Mycosis fungoides and other CTCL - and multilesional cutaneous B cell lymphomas (CBCL) is still on-going.
This monocentric dose-escalating clinical study (coded TG1042.01) was initiated in March 2002. Nine patients were enrolled in 3 successive cohorts at the Ad-IFNγ doses of $3x10^9$ vp (cohort 1), $3x10^{10}$ vp (cohort 2) and $3x10^{11}$ vp (cohort 3). Nine additional patients (cohort 4) were subsequently enrolled in order to further assess the safety profile of $3x10^{11}$ vp dose. The phase I study was then expanded in a multicentric phase I/II setting, in order to explore a larger patient population in specific cutaneous lymphoma subtypes. To this end, inclusion of 18 additional patients has been planned for the phase II step of the study to accumulate efficacy and tolerance data of Ad-IFNγ administered according to a more appropriate protocol for the disease i.e. changes in the injected lesion in case of local response, re-administration of treatment in case of relapse, multi-injection of large lesion.
The main objective was to determine the safety of repeated TG1042 intratumoral injection and determination of the Maximum Tolerated Dose (MTD). The secondary objective is to estimate the antitumoral effect of TG1042.
The main inclusion criteria were: histologic proof of CTCL or CBCL, Tumor Nodes Metastases classification (TNM) stage Ib or higher, failure of local tumor control to at least two first line treatments.

The therapeutics schedule was as follows: Intratumoral injections were to be administered to patients at weekly intervals (days 1, 8 and 15). Patients were to be evaluated at week 4. Patients without progressive disease could receive additional cycles until progressive disease was documented or toxicity requiring cessation of treatment. Patients were to be treated for up to 4 cycles in cohort 1 to 3 and up to 12 cycles in cohort 4 and in the phase II step.

Out of 36 patients enrolled 6 patients suffered from CBCL. One of them was not treated. Five local responses (3 complete responses (CR) and 2 partial responses (PR)) out of 5 evaluable patients and 3 global responses (2CR-1PR) out of the 3 evaluable patients for global evaluation were observed. The responses were seen by the end of the 2nd cycle of treatment.

In the phase I step, a total of 336 adverse events (AEs) occurred. Two hundred and seventy six AEs were considered as possibly or probably related to treatment by the investigator. AEs possibly or probably related to treatment were reported by investigator in all patients except patient n°5 (cohort 2).

All related AEs were considered by the investigators as mild or moderate except 2 AEs occurring in 2 patients (colitis occurred in patient n°2, cohort 1 and a transient grade 3 lymphopenia occurred in patient n°18, cohort 4).

Two related SAEs occurred in patient n°2 (cohort 1) and 12 (cohort 4). Except patient 12, who experienced nausea vomiting after the second injection with subsequent transient vaso-vagal loss of consciousness, no other withdrawal due to AE occurred. No death due to AE occurred.

The most commonly observed AEs related to TG1042 were classified in “Injection Site Reactions” which were experienced by all patients followed by “Febrile Disorders”, “Asthenic Conditions” and “Headache”.

2 OBJECTIVES

2.1 Primary Objective

The primary objective of this study is to evaluate the efficacy of a four-month dosing period of intra-lesional injection of TG1042 in patients with relapsing CBCL.

2.2 Secondary Objective

The secondary objective of this study is to evaluate the safety of the intra-lesional injection of TG1042 over a four-month dosing period in patients with relapsing CBCL.

3 STUDY DESIGN

3.1 Overall Study Design and Plan Description

This study is a two-step, phase II, multicenter, non comparative study of repeated intra-lesional administration of TG1042 in patients with primary CBCL.
The trial is designed as a one-sample multiple testing procedure[7].
A maximum of forty one evaluable patients (13 in the 1st step, 28 in the 2nd step) in approximately 15 centers are planned to be included into the study.

3.2 Discussion of the Study design

The purpose of the 1st step of the trial is to reject the experimental treatment from further study in the case of it is shown to be insufficiently safe and active and to accept it for further study if it is shown to be sufficiently safe and active.
The assumption of a response rate of at least 75% on TG1042 in sample size calculation is based on opinions of experts who have considered that the ratio benefit/risk for TG1042 would be medically relevant with such a response rate in this population considering the other existing systemic therapy available.

3.3 Number of Centers and Patients

Fourty one patients in approximately 15 centers are planned to be included into the study.

3.4 Patient Accrual and Duration of Study

This study is expected to start in Q2 2006 with recruitment to be completed by Q1 2008. The study is expected to be completed in Q2 2008.
It is understood that these accrual rates are based on reasonable planning expectations. The actual accrual rates will be compared to the expected rates on an ongoing basis.

4 STUDY POPULATION

Patients with primary CBCL who meet all the following inclusion and exclusion criteria, including written informed consent will be included:

4.1 Inclusion Criteria

Patients must satisfy all the following criteria for entry into the protocol:

- Primary CBCL including (according to WHO/EORTC classification 2005[1]) :
  - Primary cutaneous marginal zone B-cell lymphoma
  - Primary cutaneous follicle center B-cell lymphoma
  - Primary cutaneous diffuse large B-cell other than leg type
- Histologically consistent with primary CBCL.

- Relapse or active disease after radiotherapy or other adequate therapy if radiotherapy was contra-indicated (chemotherapy, surgical excision, interferonα, rituximab).
- Performance status of 0, 1 on the Eastern Cooperative Oncology Group (ECOG) scale (See Appendix E).
- Minimum Life Expectancy > 3 months.
- Adequate blood count: hemoglobin ≥ 10.0 g/dL; White Blood Count (WBC) ≥ 3.0 x 10^9/L; and platelet count ≥ 75 x 10^9/L.
- Adequate hepatic function: bilirubin ≤ 1.5 times the upper limit of normal and serum transaminase (SGOT and SGPT) ≤ 2.5 times the upper limit of normal.
- Adequate renal function: creatinine ≤ 1.5 times the upper limit of normal.
- Age ≥ 18 years old and < 80 years old.
- Written informed consent from patient.

4.2 Exclusion criteria

Patients will be excluded from the study for any of the following reasons:

- Primary cutaneous diffuse large B-cell lymphoma, leg type.
- Primary cutaneous intravascular large B-cell lymphoma.
- Extracutaneous involvement (sign of B-cell lymphoma on thoraco-abdominal CT scan and/or PET scan and/or on bone marrow biopsy).
- No histologic documentation of CBCL.
- History of known Human Immuno-deficiency Virus, Human Hepatitis B or C positive serology or other active systemic infections.
- Serious uncontrolled, concomitant medical disorders.
- Concomitant therapy for CBCL: surgical resection, radiotherapy, corticosteroid, chemotherapy, rituximab…(not limited listing)
- Major surgery in previous 4 weeks preceding the 1st injection.
- Pregnancy at study entry or who become pregnant during the study or women who are breast feeding.
- Males and females of reproductive potential who refuse to use adequate protection against pregnancy (intra-uterine device, hormonal contraception or diaphragm/condom and spermicide) during the conduct of the study and for three months after the last injection.
- Participation in another experimental protocol during the study period and within 4 weeks prior to the first injection.
- Patient previously included in this study.
- Non compliance with the study.

4.3 Therapy Restrictions

All prior treatments for CBCL must be stopped for 4 weeks preceding entering patient into the study.

No therapy directed against the CBCL other than that planned by the protocol is permitted while the patient is enrolled in the protocol except: emollient alone or containing menthol, polydocanol or anti-histamine given for itch control. Corticosteroid therapy (systemic and topical) and immunosuppressive / immunomodulating drugs (e.g. Cyclosporine) are not allowed. Disease

progression requiring other forms of specific antitumor therapy will be cause for discontinuation from the protocol.

5 STUDY DRUG

5.1 Characteristics and Supply

The medication under study TG1042 is made of a suspension of recombinant adenoviral particles, carrying a gene coding for the human Interferonγ (IFNγ).
TG1042 will be supplied in individual dose ampoules for single use. Each ampoule will contain $1.5x10^{11}$ vp of TG1042 in a 0.5 ml volume.
The supplies will be produced and provided free of charge by Transgene.

5.2 Packaging and Labeling

The product is supplied deep-frozen in 2 ml glass ampoules. Each ampoule is intended for single use and is shipped in a secondary plastic container. Primary labeling on the ampoules will be in English, secondary labeling on the plastic container will be in the language of the country where the study is to be performed. Secondary plastic containers are packaged in a “Safepack” which is labeled with the same information as contained on the secondary label plus the following comment: “Containing X ampoules.”

5.3 Conditions of Drug Storage and Use

Ampoules containing TG1042 or its buffer for dilution will be a frozen preparation and must be stored at or below -70°C in a freezer, under the supervision of the study pharmacist / investigator. The ampoules will be dispensed only with the written prescription of the investigator to staff that have been specifically designated to this study.

5.4 Preparation for Administration

Before injection, the final suspension must be prepared under aseptic conditions inside a laminar flow hood. The suspension must be maintained between +2°C and + 8°C after preparation and must be used within 4 hours after the syringe preparation.
TG1042 is a genetically modified organism. As such and whenever relevant, it must be handled according to national regulatory requirements.
A preparation protocol with detailed instructions will be provided by Transgene to the study pharmacist/investigator prior to the 1st administration.
All transfers of the preparation must be done using a closed container. During product manipulations, specific protective clothing must be worn (lab coat, gloves and goggles).

5.5 Waste Handling Precautions

For decontamination, either regular hospital procedures for infectious material or specific procedure provided by Transgene will be used.

In case of accidental shedding the contaminated area must be cleaned with a disinfectant active on the study drug or bleach.
In case of skin contamination with / without injury, wash immediately under tap water. Then treat the area as follows:

- Wash with soap for 5 minutes. Rinse. Then treat the area with bleach (≈ 1.4°Cl i.e. 4.5 g/l of active chlorine) for 5 minutes. Rinse again.

or:

- Wash with a solution of 4% iodine for 5 minutes. Rinse. Then treat the area with a solution of 10% iodine for 5 minutes. Rinse again.

Cover with a sterile gauze dressing, which should be appropriately discarded when removed. The injured person should receive counselling from the investigator and should then be closely followed for a period of at least 2 weeks for the development of a flu-like syndrome or other symptoms.
In case of eyes contamination, immediately irrigate the eye for 15 minutes with lukewarm water being careful not to contaminate the other eye. The injured person should receive counselling from an ophthalmologist.
In case of ingestion do not induce vomiting and call the investigator immediately. The person will be closely followed for a period of at least 2 weeks for the development of a flu-like syndrome or other symptoms.
Notify the Transgene Medical Affairs at the following hot line number: + 33 (0) 3 88 27 91 73 immediately if any accidental shedding of the product occurs.

6 TREATMENT PLAN

6.1 Treatment Administered

6.1.1 Treatment dosage

The dose of TG1042 to be administered will be $5x10^{10}$ vp per lesion. No more than 6 lesions will be treated simultaneously. The maximal total dose of TG1042 per injection day will range from $5x10^{10}$ vp (a solitary lesion) to 3 $x10^{11}$ vp (6 lesions).

6.1.2 Patients with more than 6 lesions

In case of more than 6 lesions, only 6 lesions will be injected. The designation of lesions targeted to be injected will be performed in order of preference: tumoral lesion at first, plaque lesion at second. Amongst several lesions of the same type: the largest lesion will be chosen at first.
In the event of local response and consequent disappearance of one lesion leading to the decision by the investigator to interrupt the injection of this lesion, the treatment of a lesion not previously treated but measured at baseline is authorized. However, no more than 6 lesions will be treated simultaneously.
The switch in treated lesions will be made only at D1 of a new cycle after evaluation of the previous cycle.

6.1.3 Treatment regimens

Patients will receive injections of TG1042 on day 1, 8 and 15 (injection days). The patient will receive no treatment the fourth week. The cycle length will be 4 weeks (1 cycle) and then the tumor will be evaluated for response.
In absence of premature withdrawal (see section 6.5), the 1st injection of the new cycle will be performed immediately after the tumor evaluation of the previous cycle. Thus, Day 1 of the new cycle is equivalent to Day 29 of the previous cycle.

6.1.4 Duration of treatment and observation period

In absence of premature withdrawal (see section 6.5), therapy will continue up to 4 cycles i.e. 16 weeks.
The observation period will continue up to the tumor progression.

6.1.5 Administration of the study drug

TG1042 will be administered via intra-lesional injection.
The dose injected per lesion will be $5x10^{10}$ vp (in a volume of 0.5 ml) per lesion. The injection will be done in the center of the the lesion smaller than 10 cm^2.
In case of lesion equal or larger than 10 cm^2. Two injections of a dose of $2.5x10^{10}$ vp in a volume of 0.25 ml each, will be done in the center of the 2 delimited areas.
The investigators will be provided with a procedure describing the mode of administration and a specific training will be organized.
In case of a disappearance of a lesion during a cycle, the injection of this lesion will be continue until the 3rd injection of this cycle.

6.2 Method of assigning patients to the treatment group

6.2.1 Recruitment

All patients who satisfy the main inclusion criteria (history of CBCL, relapse after radiotherapy or other standard therapy if radiotherapy is contra-indicated) will be considered as pre-selected patients. Every pre-selected patient will be screened for inclusion into the study.
The recruitment process will be documented via a “Screening log”, listing the initials and dates of birth of all patients pre-selected for the study (see Appendix D). The patient’s study number when eligible will be recorded on the screening log. The reason(s) for the non-inclusion when appropriate will be recorded on the screening log.

Supprimé: Appendix DAppendix DAppendix D

6.2.2 Randomization

Not applicable.

6.2.3 Dose modifications

No dose modifications are permitted.

6.3 Safety stopping rules

Dose limiting toxicity (DLT) is defined as any grade 3 or higher toxicity (according to NCI/CTCAE) considered as both related to TG1042 and clinically significant by the Data Safety Monitoring Committee (DSMC). Grade 3 or 4 fever and grade 3 transient lymphopenia are not considered as limiting toxicity.
Study should be stopped if DLT is observed in:

- Greater than one of the first 3 patients treated with TG1042.
- Greater than one of the first 6 patients treated with TG1042.
- Greater than two of the first 9 patients treated with TG1042.
- Greater than four of the first 13 patients treated with TG1042.
- Thirty three percent or more of subjects treated with TG1042 at any time in the phase 2nd step of the study.

6.4 Blinding/Unblinding

Not applicable

6.5 Premature withdrawal of patients

The patient’s participation in the protocol must be terminated under any of the following circumstances:

- Pregnancy during the study.
- Patient’s request at any time for any reason.
- Tumor progression as defined in section 8.3.
- Physician determination that patient’s further participation in the protocol is not in the patient’s best interest.
- At the determination of the DSMC (for example in case of new fact or toxicity issue regarding the study drug).
- Patients who have severe unacceptable toxicity associated with the study drug.

For any discontinuation, the investigator will obtain all the required details and document the date of and the reason for the discontinuation in the Case Report Form (CRF). In case of treatment cessation not related to disease progression the patient will be followed per protocol until documentation of progressive disease.
If the reason for discontinuation was an AE, the specific event or the main laboratory abnormality will be recorded in the CRF. The investigator will make thorough efforts to document the outcome.

6.6 Replacement policy

Patients who will be considered as non evaluable (see definition page 8) will be replaced.

7 STUDY VISITS AND PROCEDURES

A flow-chart shown in Appendix A summarizes the evaluations to be performed and their time points.

Supprimé: Appendix AAppendix AAppendix A

7.1 Assessments to be performed

- Complete medical history.
- Interim medical history of events occurring since the last visit.
- Physical examination: examination of the major organ systems, including height and weight.
- Vital signs: body temperature, heart rate, respiratory rate and blood pressure.
- Safety assessment: reporting of Pre-Drug Events (PDEs) and documentation and assessment of all AEs since the previous assessment (see section 9).
- Tumor evaluation and imaging (see section 8).
- Complete Blood Count including hemoglobin, WBC, differential and platelets count.
- Standard laboratory parameters:
  - Liver chemistry: bilirubin, alanine amino-transferase, aspartate amino-transferase, alkaline phosphatase, prothrombin time.
  - Serum creatinine.
- Pregnancy test for woman.
- Proteomic and transcriptomic analysis.
- Electrocardiogram (ECG).
- Abdomino-thoracic CT scan and/or PET scan.
- Bone Marrow biopsy (if not previously performed since relapse).
- Biopsy of a representative CBCL lesion with immuno-histochemistry.
- Biopsy of a representative node (if necessary).

7.2 Baseline: On days -14 to 0

Baseline evaluation (except biopsies) will be performed within the 2 weeks prior the 1st injection and include:

- Complete medical history, physical examination and vital signs.
- Tumor evaluation and imaging.
- Complete blood count.
- Pregnancy test for woman.
- Proteomic and transcriptomic analysis.
- Standard laboratory parameters.
- ECG.
- Abdomino-thoracic CT scan and/or PET scan.
- Bone marrow biopsy within the 4 weeks preceding the 1st injection if not previously performed since relapse and/or in case of increase of lymphocytes blood count or other signs of blood involvment.
- Biopsy of a representative CBCL lesion within the 4 weeks preceding the 1st injection.

- Biopsy of a representative node in case of clinical abnormality (see section 8.1.4).

7.3 While enrolled on the protocol

7.3.1 Days 1, 8 and 15 (injection days) of each cycle:

Before injection:

- Interim medical history since the last visit, physical examination and vital signs.
- Safety assessment: reporting and documentation of all AEs (PDEs at day 1 of the 1st cycle) since the previous assessment.
- Complete blood count.

After injection:

- Physical examination 1 hour after the injection.
- Vital signs: the patient will be closely monitored hourly for a minimum of four hours after the first injection and two hours after subsequent injections of the study drug.

7.3.2 Day 29 (day of tumor evaluation) of each cycle:

In case of decision of continuation, the 1st injection of the new cycle will be administered the day of evaluation of the previous cycle. Thus Day 1 of the new cycle will be equivalent to Day 29 of the prior cycle. At this visit, the following examinations will be done:

- Interim medical history since the last visit, physical examination and vital signs.
- Safety assessment: reporting and documentation of all AEs since the previous assessment.
- Tumor evaluation and imaging will be done in order to have the results available prior to the first injection of the subsequent cycle.
- Standard laboratory parameters.

In addition, at Day 29 of the last cycle (completed or not) in case of discontinuation:

- Complete blood count
- ECG.
- Abdomino -thoracic CT scan and/or PET scan in case of response.
- Biopsy of a skin area previously involved in case of complete response.
- Biopsy of a representative node in case of clinical abnormality (see Section 8.2.2).

7.4 End-of-treatment visit (to be conducted 4 weeks after Day 29 of the last cycle):

The following examinations will be done:

- Interim medical history since the last visit, physical examination and vital signs.
- Safety assessment: reporting and documentation of all AEs since the previous assessment.

7.5 Post-Treatment Follow-Up

If no tumor progression occurs during the treatment period, a post-treatment follow-up will be performed until tumor progression on a monthly basis during the first 6 months and then on a quarterly basis for one year. This visit will include:

- Interim medical history since the last visit, physical examination and vital signs.
- Tumor evaluation and imaging.

8 TUMOR EVALUATION

8.1 Baseline Evaluation

The baseline evaluation will be performed as closely as possible to the first injection and no more than 2 weeks before the first injection.

8.1.1 Skin lesion evaluation

Skin lesion evaluation will be performed by the local, independent evaluator, dermatologist who will be not involved in the management of the patient. A specific training for the evaluator will be organized in order to standardize the evaluation process. As far as possible, the same patient will be evaluated by the same evaluator.
All lesions will be identified and categorized as follows:

- Plaque: palpable infiltrated lesion.
- Tumor: nodular lesion.

All lesions will be measured by use of a ruler or callipers in two diameters. All measurements will be recorded in metric notation. For each lesion, the longest diameter will be the first recorded. The longest diameter perpendicular to the first will be the second diameter. The product of these two diameters will be calculated and are defined as the "product" of the lesion.
The sum of products (SP) of all lesions will be calculated according to the following formula:
SP = Σ (plaque surface) $_+$ 2xΣ (tumor surface)

8.1.2 Central lymph nodes evaluation

An abdomino-thoracic CT scan and/or PET scan will be performed at baseline to exclude central lymphoma with secondary skin involvement.

8.1.3 Hematological evaluation

A bone marrow biopsy will be performed at baseline if not previously undertaken to exclude hematological involvement.

8.1.4 Peripheral lymph nodes evaluation

Any peripheral lymph nodes draining cutaneous lesions and cervical, supraclavicular, epitrochlear, axillary and inguinal nodes groups will be systematically explored by physical examination. Lymph nodes palpable and abnormally enlarged (diameter >1.5 cm) will be considered as clinical adenopathy.
In case of clinical abnormality leading to suspicion of a tumoral involvement, a representative biopsy will be performed whenever possible.

8.1.5 Visceral evaluation

If visceral involvement is clinically suspected, any appropriate exploration will be undertaken in order to exclude visceral extent.

8.1.6 Quality of Life evaluation

Patient will be evaluated with the Dermatology Life Quality Index (DLQI) questionnaires (see Appendix B). DLQI is the first published and well validated dermatology specific health-related quality of life[8]. It consists of ten items concerning symptoms and feelings, daily activities, leisure, work and school, personal relationship and treatment. The questionnaires will be completed by the investigators.

Supprimé: Appendix BAppendix BAppendix B

8.2 On treatment evaluation

Evaluation will be performed at day 29 of each cycle, at the last visit and at each monthly follow-up visit up to tumor progression or death whichever occurs first in case of absence of progression during the treatment period.

8.2.1 Skin lesion evaluation

The on-treatment evaluations will be performed, as far as possible, as they were for the baseline evaluation. The ratio of the SP of each lesion will be calculated taking as reference the baseline assessment.

8.2.2 Lymph node evaluation

Peripheral lymph nodes draining cutaneous lesions and cervical, supraclavicular, epitrochlear, axillary and inguinal nodes groups will be systematically explored by physical examination. Lymph nodes palpable and abnormally enlarged (diameter > 1.5 cm) will be considered as clinical adenopathy. In case of clinical abnormality leading to suspect a tumoral involvement, a representative biopsy will be performed whenever possible.

8.2.3 Visceral evaluation

If a visceral involvement is clinically suspected, any appropriate exploration will be undertaken in order to exclude visceral extent. Extracutaneous metastasis should be confirmed by biopsy and will be considered as a progression criterion.

8.2.4 Quality of Life evaluation

Patient will be evaluated at day 29 of each cycle.

8.3 Response Criteria

Response criteria for tumor response are clinically defined (taking as reference the baseline evaluation) as follows:

- Complete response (CR) is defined as the clinical disappearance of all lesions.
- Partial response (PR) is defined as the clinical disappearance of at least half of all lesions.
- Minor response (MR) is defined as a decrease of at least 50% in the SP of all lesions with the clinical disappearance of less than half of all lesions.
- Stable disease (SD) is defined as any response that did not meet the criteria for CR, PR, MR or PD.
- Progression (PD) is defined as an increase of at least 25% in the SP of all lesions. Appearance of a new cutaneous lesion, lymph nodes, hematological or visceral involvement will be considered as a PD.

Tumor response (complete or partial) will be confirmed by repeated assessment that will be performed 4 weeks after the criteria for response are first met.

8.4 Tumor Imaging

At each tumor evaluation overall photographs (total hemi-body anterior and posterior) and photographs of every lesion will be taken serially. Photographs will be taken with the patient in a consistent pose and with a consistent technique with respect to light, angle and distance.
Transgene will provide a standardized digital photographic system, procedure and training (see Appendix C).

Supprimé: Appendix CAppendix CAppendix C

9 ASSESSMENT OF SAFETY

The condition of the patient will be monitored throughout the study.
Overall incidence of AEs and SAEs will be evaluated.

9.1 Definitions

9.1.1 Adverse Event

Any untoward medical occurrence in a patient or clinical investigation subject administered a pharmaceutical product and which does not necessarily have a causal relationship with this treatment. An AE can therefore be any unfavorable and unintended sign (including an abnormal laboratory finding), symptom, or disease temporally associated with the use of a medicinal (investigational) product, whether or not related to the medicinal (investigational) product. An AE is therefore regarded as such only if it starts after the first administration of the study drug.

9.1.2 Pre-Drug Event

Any event occurring before the first study drug administration, i.e. between the patient signature of the informed consent form and the first administration of the study drug.

9.1.3 Laboratory abnormality

A laboratory abnormality is reported as an AE if it is out of range and considered by the investigator as clinically significant (i.e. with clinical manifestations or requiring treatment or clinical management) and confirmed by a repeated measurement (if relevant).

9.1.4 Other significant Adverse Event

Any event and any laboratory abnormality that led to an intervention, including withdrawal / dose reduction of study drug or significant additional concomitant therapy other than those reported as SAE and that are considered by Transgene or the investigator to be of special interest because of clinical importance.

9.1.5 Serious Adverse Event

Any untoward medical occurrence that at any dose:

- Results in death. The death of a patient is not per se an AE but an outcome. “Death” will be considered as a SAE only in case of “unexplained death" when no cause is identified. The event that resulted in a fatal outcome will be determined.
- Is life-threatening. This term refers to an event in which the patient was at immediate risk of death at the time of the event; it does not refer to an event that hypothetically might have caused death if it was more severe.
- Requires inpatient hospitalization or prolongation of existing hospitalization. The hospitalization is an action taken to treat the event. It will not be reported as a SAE, but the AE leading to hospitalization. Hospitalization for diagnosis or planned treatment procedures without AE will not be reported as a SAE.
- Results in persistent or significant disability/incapacity. The disability is a substantial disruption of a person’s ability to conduct normal life functions.
- Is a congenital anomaly/birth defect.
- Is an important medical event that may not be immediately life-threatening or result in death or hospitalization, but may jeopardize the patient and may require medical or surgical intervention to prevent one of the outcomes listed above (example: intensive treatment in an emergency room or at home for bronchospasm, convulsions that do not result in hospitalization…). Medical and scientific judgment will be exercised in deciding whether some events will be considered as serious because their quick reporting to Transgene may be of interest for the overall conduct of the study.

9.1.6 Serious Pre-Drug Event

Serious Pre-Drug Event is defined as any serious event occurring before the first study drug administration, i.e. between the patient signature of the informed consent form and the first study drug administration.

9.1.7 Overdose

An overdose is an administration of a study drug at a higher dose than the highest dose already tested in clinical studies ($3x10^{11}$ vp by injection) or higher than known therapeutic doses.

9.2 Intensity, relationship and outcome evaluation

9.2.1 Intensity

The intensity of the clinical AE is graded according to the Common Toxicity Criteria for Adverse Event (CTCAE) version 3.0 (12 December 2003) which is provided to the investigators. Should an event be missing in the CTCAE, the following 3 point scale is used:

- Mild: Discomfort noticed, but no disruption of normal daily activity.
- Moderate: Discomfort sufficient to affect normal daily activity.
- Severe: Inability to work or perform normal daily activity.

The correspondence between the two scales is as follows:

CTCAE	3 point scale
1	Mild
2	Moderate
3	Severe
4	
5	

9.2.2 Relationship to the study drug

The relationship is evaluated as follows:

- Unrelated: There is evidence of relationship to a cause other than the study drug. Does not meet criteria listed under unlikely, possible or probable.
- Unlikely: Does not follow a reasonable temporal sequence from administration. Is most likely produced by the patient's clinical state or by environmental factors or other therapies administered.
- Possible: Follows a reasonable temporal sequence from administration. Is not likely produced by the patient's clinical state or by environmental factors or other therapies administered.
- Probable: Follows a reasonable temporal sequence from administration. Clear-cut temporal association with improvement on cessation of test drug. Reappears upon rechallenge.

9.2.3 Outcome

The outcome is rated as follows:

- Recovered.
- Not recovered.
- Recovered with sequelae (to be specified on comment page).
- Fatal.
- Unknown.
- Worsening.

Note on "fatal": this outcome is to be used only for the event leading to death. The outcome of all other events at the time of the death must be reported. The outcome of ongoing ones is reported as "not recovered".
Note on “worsening”: this outcome is used when a PDE worsens and becomes an AE or when an AE worsens (in these situations, a second line is completed on the AE page documenting the new status of the event).

9.3 Pre-Drug Event management

9.3.1 Reporting in Case Report Forms

Any PDE (see definition section 9.1.2) directly observed or mentioned by the patient will be reported by the investigator or designee on the page "Events occurring before the first study drug administration" of the CRF. The following items must be documented:

- Nature of the event with self explanatory and concise medical terminology (indicate a diagnosis or syndrome instead of symptoms).
- Date of onset and date of end (i.e. dates when the event starts and ends and not dates when the investigator is informed).
- Outcome.
- Intensity.
- Action taken regarding the study.
- Action taken regarding the event.
- Evaluation of seriousness.

9.3.2 Follow-up

If those events are not resolved before the first study drug administration they will be followed until resolution or the last study visit planned by the protocol:

- PDE with no change will not be considered as AE. The outcome is documented when known. If a PDE is still ongoing at the last study visit, the status at this time will be documented.
- PDE that worsens after the first study drug administration will be considered as AE and managed as follows:
  - On the CRF page "Events occurring before the first study drug administration", the line of the PDE is completed with the outcome ticked as “worsening”.

- On the CRF page “Adverse events”, a new event is reported and managed as described in section 9.4. The wording of the event will include "worsening of" or something similar.

It is possible that, due to its nature or intensity, a PDE could delay the first study drug administration or lead to patient withdrawal. This will be documented on the CRF "Events occurring before the first study drug administration" pages in the column “Action taken regarding the study”.

9.3.3 Documentation

PDEs will be reported in the patient source document with at least the nature, the start date and the treatment (if applicable). PDE pages of the CRF will be signed by the investigator once all lines of all PDEs reported are fully completed.

9.4 Adverse Event management

9.4.1 Reporting in Case Report Form

At each visit, any AEs directly observed or mentioned by the patient will be reported by the investigator or designee on the page “Adverse Events” of the CRF. The following items must be documented:

- Nature of the event with self explanatory and concise medical terminology (indicate a diagnosis or syndrome instead of symptoms).
- Date of onset and date of end (i.e. dates when the event starts and ends and not dates when the investigator is informed).
- Outcome.
- Intensity.
- Relation to study drug.
- Action taken regarding the study drug.
- Action taken regarding the event.
- Evaluation of seriousness.

AEs requiring therapy must be treated with recognized standards of medical care to protect the health and well being of the patient. Any treatment given will be reported on the page "Concomitant medication" of the CRF.

Appropriate resuscitation equipment and medicines must be available to ensure the best possible treatment of an emergency situation.

9.4.2 Grade 3 to 4 Adverse Event

Grade 3/4 AEs and/or any other significant AE related to the study drug which do not qualify as a SAE will be further documented on "additional information on adverse events" CRF page.

9.4.3 Follow-up

AEs must be followed up at least until the last visit planned by the protocol. The outcome is documented on the CRF AE pages.

Some AEs must be followed until they are resolved or stable (i.e. may be after the last visit planned by the protocol):

- AEs with a possible/probable relationship with the study drug.
- AEs leading to withdrawal from the study.
- Any other significant AE.

9.4.4 Documentation

AEs will be recorded in the source document with at least the nature, the start date, the end date, the outcome and the treatment (if applicable) of the event. AE pages of the CRF will be signed by the investigator once all lines of all AEs reported are completed.

9.5 Serious Adverse Event management

9.5.1 Reporting to Transgene

Any SAE occurring during the course of a study, i.e. between the first study drug administration and the end of treatment visit, irrespective of the treatment received by the patient MUST be reported to Transgene. within ONE WORKING DAY of occurrence or knowledge of the event. The investigator must complete and fax a "Serious Adverse Event Form" which will be provided by Transgene to the investigator.

	EUROPE	USA
	Safety Officer	Clinical Operations
Name	I. DIDILLON	A. DERBIJ
Phone number	(33)(0)3.88.27.91.73	+ (301) 816-5421
Fax number	(33)(0)3.88.27.91.41	+ (301) 816-5439
Emergency 24-hour telephone number	(33)3.88.27.91.73	+ (301) 318-5118

An investigator's designee may complete the "Serious Adverse Event Form". However, the SAE form should be signed by the investigator. If this is not possible within one working day, the form is sent to Transgene with the designee's signature. Investigator's signature must be obtained as soon as possible, as well as his/her evaluation of the relationship to the treatment. The signed form must be faxed to Transgene immediately.
The "Serious Adverse Event Form" should be completed in English.

9.5.2 Special cases

The following events will be documented, reported and followed-up in the same way as a SAE:

- Serious PDEs.
- Overdose (even if no toxic effects are observed).
- Pregnancy (to be followed up in the same way as a SAE until the final outcome of the pregnancy and knowledge of the new-born medical status).

9.5.3 Follow up

It is possible that at the time of the event information is missing. This must then be forwarded as soon as possible to Transgene, on a new SAE form with the box "follow-up" ticked. Transgene may also ask for information as needed.
All SAEs will be followed-up by the investigator until final outcome is known. Follow-up information is sent to Transgene by the investigator on a "Serious Adverse Event Form" with the "follow-up report" box ticked. The frequency of the follow-up is up to the investigator i.e. as soon as relevant new information becomes available up to the final outcome. New information is sent within one day of knowledge.

9.5.4 Post study Serious Adverse Event

Any SAE occurring after the end of treatment visit and that is considered as possibly or probably related to the study drug by the investigator, must be reported to Transgene, documented and followed-up as described above.
If the end of treatment visit is performed less than 28 days after the last study drug administration, all SAEs occurring between the end-of-treatment visit until 28 days after last study drug administration will be considered as SAE and reported to Transgene, whatever their relationship to study drug is according to the investigator.

9.5.5 Reporting to Regulatory Authorities / Gene Therapy Bodies / Ethics Committees

Transgene is responsible for notifying of Health Authorities for all study SAEs in accordance with local regulation. Gene Therapy Bodies will be informed as locally required.
The investigator or Transgene is responsible for informing the Independent Ethics Committee (IEC) / Institutional review Board (IRB) in a timely manner and in accordance with local procedures.

9.5.6 Information to investigator

When a serious, unexpected, probably or possibly related event has been reported to the Health Authorities, Transgene will inform all other investigators working in this study as well as those working with the same study drug in other studies.

9.5.7 Documentation

All SAEs will be reported in the CRF using the same information as the one reported on the SAE form and in source documents as described in §9.4. Copies of SAE form will be filled in the Investigator Site File with copies of any correspondence with the IEC/IRB and copies of reporting letters and/or faxes (with reporting forms) to Health Authorities and Gene Therapy Regulatory Bodies.

9.6 Laboratory values, vital signs, physical findings and other safety data

In the event of clinically relevant laboratory abnormality, the test will be repeated (if relevant) and followed-up until it has have returned to normal except if an adequate explanation is found. Clinically relevant abnormal findings following vital signs measurement or physical examination will be reported as an AE.

10 STATISTICAL METHODS PLANNED AND SAMPLES SIZE

10.1 Determination of Sample Size

The purpose of the trial is to reject the experimental treatment from further study if it is shown to be insufficiently active and to accept it for further study if it is shown to be active. The study is designed as a two-stage 'optimum' phase II trial with the following assumptions[7]:

- The inactivity cut-off is chosen equal to 50%, the activity cut-off equal to 75%. Hence the hypotheses of interest are H_0: $r \leq 50\%$ against H_A: $r \geq 75\%$, where r is the response rate
- The type I error rate (α, probability of accepting an insufficiently active treatment, a false positive outcome) is set to 5%
- The type II error rate (β, probability of rejecting an active treatment, a false negative outcome) is set to 10%

Under these assumptions, an optimal design consists of the following two stages:

Treat 13 evaluable patients:

- if at most 7 responses are observed, stop the trial and declare the drug insufficiently active
- otherwise continue patient entry:

Treat additional evaluable patients up to a total of 41

- if at most 25 responses are observed, declare the drug insufficiently active
- if at least 26 responses are observed, declare the drug active

With this design, if the response rate is truly equal to 50%, the expected sample size is 27 evaluable patients and the maximum sample size is 41 evaluable patients.

10.2 Study Endpoints

10.2.1 Primary Endpoint

- Response rate. The primary endpoint will be the response rate which includes complete, partial and minor responses. The tumor response is defined as the best tumor response recorded from the 1st cycle evaluation until the last cycle evaluation. To be taken into account, tumor response (complete, partial or minor) will be confirmed by repeated assessment that will be performed 4 weeks after the criteria for response are first met.

10.2.2 Secondary Efficacy Endpoints

- Duration of response. The duration of response is measured from the time that measurement criteria are first met for CR, PR or MR (whichever status is recorded first) until the first date of PD is objectively documented or the last visit if no progression occurred (censored data).

- Time to progression. Time to progression is measured from the date of inclusion until the first date of PD is objectively documented or the date of the last visit if no progression occurred (censored data).

- Quality of life according to Dermatology Life Quality Index

10.2.3 Secondary Safety Endpoints

- Incidence of AE

- Incidence of SAE.

10.3 Disposition of Patients

All patients who satisfy the main inclusion criteria (history of CBCL, relapse after chemotherapy) will be registered as pre-selected patient and reported in the screening log. All of them who failed to meet inclusion criteria will be reported as screening failure in the study report with the reason(s) for their non-inclusion.
All patients included, will be considered in the study report. All post-inclusion discontinuations will be summarized by main reason for discontinuation.

10.4 Protocol Deviations

Any protocol deviation will be documented in the CRF and discussed with the investigator on a case by case basis.
The following circumstances will be considered as major entry criteria violation:

- No histological documentation of CBCL.
- No prior radiotherapy (or other standard therapy if radiotherapy is contra-indicated).

Included patients who will meet one of these criteria will be defined as “wrongly included” patient.

10.5 Statistical and Analytical Plans

A comprehensive statistical analysis plan describing all analyses will be developed and filed before the database is locked.

10.5.1 Efficacy Data

An exact 95% confidence interval for the observed response rate will be calculated. Response rates will be compared by a Chi-squared test, level of significance is fixed to 0.05 two-sided.

Duration of response, time to progression will be estimated by the Kaplan-Meier methods, plotted as curves and compared by log-rank test.
The need to correct for multiplicity will be addressed in case that decision is to be based on the results of statistical tests.

10.5.2 Safety Data

The safety data will be classified according to their intensity and relationship to the study drug. All AEs, laboratory test changes, etc. will be identified, coded with the Medical Dictionary for Regulatory Activities, classified and analyzed, as appropriate. The number of patients with each AE will be displayed by decreasing frequency of occurrence and body system classes. All AEs occurring after the initiation of the study treatment, including events likely to be related to the underlying disease or likely to represent concomitant illness will be reported, including events present at baseline which worsened during the study. Written narratives will be provided for all serious, unexpected or other significant AEs that are judged to be of special interest because of their clinical importance.
Supplementary analysis using clinically significant cut-off points for the laboratory test results will also be performed, if appropriate.

10.6 Data Sets Analyzed

10.6.1 Efficacy analysis

The efficacy analysis will be performed on both "intention to treat" and "per protocol" principles. The main analysis will be based on "intention to treat". "Per protocol" analysis will be secondary analysis.
Intention to treat analysis is performed:

- on every included patient (non evaluable patient for whom the data is not available for on-site evaluation of the tumor response will be considered as PD),
- based on the on-site evaluation of the tumor response by the evaluator.

Per protocol analysis is performed:

- on all patients who:
  - are not wrongly included (see definition in section 10.3),
  - are treated with at least 3 injections of TG1042 (administered within at most 4 weeks),
  - are evaluable (for whom the data is available for on-site evaluation of the tumor response).
- Based on the on-site evaluation of the tumor response by the evaluator.

10.6.2 Safety analysis

The safety analysis will be performed on all patients:

- who are treated with at least 1 injection,
- for whom the parameter is available.

11 CHANGES IN THE CONDUCT OF THE STUDY

11.1 Protocol amendments

No change will be implemented to this protocol without being agreed by the investigator(s) and Transgene prior to its implementation. All protocol amendments will be issued by Transgene, signed and dated by the investigator. It will be submitted for consideration to the Health Authorities and approving Independent Ethic Committee(s) (EC(s))/Institutionnal Review Board(s) (IRB(s)) by the investigator or Transgene according to local regulations.
Ethical approval will be required for any change to the protocol which could significantly affect the safety of patients, the scope of the investigation or the scientific quality of the study (e.g. any increase in drug dosage or duration of exposure of individual patients to the study drug beyond that of the current protocol, or any significant increase in the number of patients under study; any significant change in the design of the protocol such as the addition or dropping of a control group, the addition of a new test or procedure that is intended to improve monitoring for, or reduce the risk of, a side effect or AE, or the dropping of a test intended to monitor safety).
Other changes will be provided to IEC(s)/IRB(s) for information only.
IEC(s)/IRB(s) approval will be obtained before implementation of change(s) except where necessary to eliminate immediate hazards to the patients or when the change(s) involves only logistical or administrative aspects of the study.

11.2 Premature study termination

Both the investigator and Transgene reserve the right to terminate the study at any time. Should this become necessary, the procedures will be agreed upon after consultation between the two parties. In terminating the study, Transgene and the investigator will assure that adequate consideration is given to the protection of the patient. Transgene will notify to the Health Authorities and the IRB/IBC of the premature study termination according to local regulations.

12 ETHICAL CONSIDERATIONS

12.1 Independent Ethics Committee/Institutional Review Board

Before starting the study, this protocol, the written patient information sheet and informed consent form and any other documents specifically requested must be submitted to the appropriate EIC/IRB.
The IEC/IRB approval must be obtained in writing with the list of members having participated in the meeting before the study may begin.
In addition, the IEC/IRB approval must be obtained for any amendment to the informed consent documents or to the protocol according to section 11.1.

12.2 Informed consent

The investigator will obtain a voluntary written informed consent from each patient after an appropriate explanation of the aims, methods, anticipated benefits, potential hazards and any

other aspect of the study relevant for the patient's decision to participate. Consent forms and all verbal study related information must be in a language fully comprehensible to the prospective patient.
Patients will be informed that they are free not to participate in the study and that they may withdraw consent to participate at any time. They will be told which alternative treatments are available if they refuse to take part and that such refusal will not prejudice future treatment.
Patients will be informed that their records, including medical history, may be examined by competent authorities and authorized persons but that personal information will be treated as strictly confidential and will not be publicly available.
A written "patient information sheet" will be given to each patient to complete the verbal information. This written form will be reviewed orally with the patient. Patient must be given sufficient time to review this information and opportunity to inquire about details of the study.
The "patient information sheet" will explain that the data collected for this study will be stored in a computer database, with confidentiality maintained in accordance with national data legislation. All data computer processed will be identified by patient initials and patient number only.
Informed consent shall be documented by the use of a written consent form approved by the IEC/IRB and signed and dated by the investigator and the patient before any exposure to a study-related procedure, including screening tests for eligibility. A copy of the signed informed consent form must be given to the patient. The original hard copy will be filed in the investigator site file.

12.3 Confidentiality of patient data

The investigator must assure that patients' anonymity is maintained and that their identities are protected from unauthorized parties. On CRFs or other documents collected by Transgene or its representative, patients will not be identified by their names, but by an identification code system which will consist of their initials and a number.
Only the investigator will keep a patient identification log showing codes, names and addresses of all study participants. A copy of this log without names and addresses will be filed at Transgene at the end of the study.
In the USA, confidentiality of patient's medical records will be maintained in accordance with HIPAA regulations. As a part of the regulatory approval process at the site, the investigator will consult with the local hospital administration regarding the language that the site should use.

12.4 Data Safety Monitoring Committee

12.4.1 Responsibility

The DSMC responsibilities are included the following:

- Protect the safety of the study patients.
- Consider factors external to the study when relevant information becomes available, such as scientific or therapeutic developments that may have an impact on the safety of the patients.
- Make recommendations to the sponsor concerning continuation, termination or other modifications of the trial based on the safety stopping rules defined in section 6.3.

- Make recommendations to the sponsor concerning termination or the continuation in the 2nd step of the trial based on the observed response rate in the 1st step.
- Assist the sponsor by commenting on any problem with safety of the patients.

12.4.2 Membership

The DSMC will consist of 5 voting members. The members will be recommended by Transgene and approved by the study coordinator. Members will be appointed by Transgene. Membership consists of persons completely independent of the investigators who have no financial, scientific, or other conflict of interest with Transgene. The DSMC includes:

- Two experts of the fields of CBCL.
- One expert of the fields of safety management designated as Safety Manager.
- One expert of the fields of viral vector-based therapy.
- One expert in biostatistics.

The chairperson will be selected by Transgene. He/She is responsible for overseeing the meetings, developing the agenda in consultation with the Transgene CPM (or his/her designee). The Safety Manager will be the contact person for the DSMC. Transgene CPM (or his/her designee) will serve as ex-officio member. Transgene shall provide the logistical management and support of the DSMC.

12.4.3 Board Process

Meetings may be convened as conference calls as well as in person, except for the initial meeting.

The initial meeting will take place face-to-face before initiation of the trial to discuss the protocol and to establish guidelines to monitor the safety data. Transgene CPM (or his/her designee) will prepare the agenda to address the review of manual of operating procedures, reporting of AEs and SAEs, stopping rules etc.

Meetings of the DSMC will be held at the call of the Chair with prior approval of Transgene CPM (or his/her designee). Meetings will be held at least after the completion of the treatment of the 13th and the 27th evaluable patients. Every meeting will be attended by the Safety Manager and at least 2 experts which constitutes a quorum and by the Transgene CPM (or his/her designee). Meetings may be attended by the study coordinator when appropriate. Meetings shall be closed to the public because discussions may address confidential patient data.

An emergency meeting of the DSMC may be called at any time by the Chairperson or by Transgene if questions of patient safety arise.

Each meeting will conclude with a decision to continue or to terminate the study. This recommendation will be made by formal majority vote. A termination decision will require the vote of all DSMC members. In the event of a split vote, a minority report will be contained within the regular sponsor report.

12.4.4 Meeting Reports

On a scheduled basis (as agreed upon by the DSMC) safety data will be communicated to all DSMC members or to the designated Safety Manager in a “meeting package”. Any concerns

noted will be brought to the attention of the Chair or designated Safety Manager who will take appropriate action.
The meeting package will be prepared by the biostatistician members of the DSMC and distributed to the DSMC at least 10 days prior to a scheduled meeting. The contents of the package are determined by the DSMC. Additions and other modifications to these packages may be directed by the DSMC on a one-time or continuing basis. Copies distributed prior to and during a meeting are collected by the biostatistician following the meeting.
A formal report from the Chair of session will include sufficient information to support the rationale for any recommended changes. In the event of a split vote, a minority report will be contained within the regular DSMC report. The report will be sent to the full DSMC within 2 weeks of the meeting. Once approved by the DSMC, the Chair will forward the approved minutes of the report to Transgene within 3 weeks following each meeting.
In the event of a termination recommendation, the Chair will transmit such a recommendation to the sponsor as rapidly as possible, by immediate telephone and fax if sufficiently urgent.

12.4.5 Confidentiality

All materials, discussions and proceedings of the DSMC are confidential. Members and other participants in DSMC meetings are requested to maintain confidentiality.

13 REGULATORY CONSIDERATIONS

13.1 Regulatory considerations

This study will be conducted in accordance with:

- The Declaration of Helsinki adopted by the World Medical Association.
- The ICH (International Conference on Harmonization) Good Clinical Practice (GCP) guidelines and
- The local regulatory requirements.

13.2 Regulatory approval / authorization

The regulatory permission for conducting the study will be obtained in accordance with local regulatory requirements. Additional approvals will be obtained from the national gene therapy and viral safety committees, as required. All approvals must be obtained before a patient is exposed to a study-related procedure, including screening tests for eligibility.

13.3 Investigators obligations

Before the study starts, the investigator shall forward to Transgene with his/her curriculum vitae, FDA 1572 form fully completed and shall complete a list giving the names, functions and authorized activities of all persons who will exercise any kind of responsibility in carrying out of the study.

The investigator ensures the quality of the study through strict observance of ICH GCP and of the protocol. Investigator must ensure that the study has been approved by all required institutional ethics and scientific committees prior to enrolling patients and on an ongoing basis as locally required. Investigator is required to obtain written inform consent from each patient prior to the initiation of the screening process.

13.4 Insurance

Transgene certifies having taken out a civil liability insurance policy covering liability with regard to the participants in this study.

14 QUALITY CONTROL AND QUALITY ASSURANCE

14.1 Source data and documents

Source data are all information available in original source document or certified copies of source document of any clinical findings, observations, or other activities that are necessary for the reconstruction and evaluation of the study.
For each patient enrolled, the investigator will indicate in the source documents that the patient participates in this study and will record the appropriate information. This will include (non-exhaustive list): patient name, date of birth, sex, medical history, information that the patient is included in the study, visit dates, product administration, primary evaluation criteria, nature of AEs with date of start and related treatment.
The investigator will permit study-related monitoring, audit(s) and regulatory inspection(s), with a direct access to all the required source documents each time it is necessary provided that patient confidentiality is protected. The extent of source data verification performed by the monitor is 100% of critical data (regarding eligibility criteria, treatment administration, safety and efficacy assessment) and 20% of not critical data to be verified per CRF.
Source documents will be preserved for the maximum period of time requested by local recommendation or ICH, whichever occur the last.

14.2 Periodic monitoring

The monitor will contact and visit the investigator periodically to evaluate the study progress and the compliance of the study site with GCPs, regulations and the study protocol as well as to verify and collect data reported in the CRF. The investigator as well as any study staff member will co-operate with the monitor to ensure that any problem that may be identified is resolved.
For this study, the average frequency of the monitoring visits is intended to be approximately every 6 weeks during the period while a patient will be treated with the first visit occurring as soon as possible after the first patient inclusion. Intervals may be adjusted according to patient accruals or site performance.

14.3 Audit and inspection

The investigator will make all study-related source data and documents available to a quality assurance auditor mandated by Transgene, or to domestic or foreign regulatory inspectors, after appropriate notification. The main purposes of an audit or inspection are to confirm that the rights and well-being of the patients have been adequately protected and that all data relevant for the evaluation of the study drug have been processed and reported in compliance with ICH GCP and applicable regulatory requirements.

15 DATA HANDLING AND RECORD KEEPING

15.1 Investigators information

The investigator will be kept informed of the methods for relevant ratings and completion of CRFs and of important information that relates to the safe use of the study drug as the study proceeds.
The site personnel, e.g. residents, nurses, laboratory technicians and any other personnel providing care to the patients or handling biological specimen will be informed of the characteristics of the study drug and receive safety instructions. The staff training will be documented in the investigator site file and a copy of this document will be provided to Transgene.

15.2 Case report forms (CRF)

For each patient enrolled, a CRF must be completed, in English, by the investigator or designee and signed by the investigator. This does not apply to records for those patients who fail to complete the screening period. If a patient is withdrawn from the study, the reason must be noted in the CRF. If a patient is withdrawn from the study because of a treatment-limiting AE, thorough efforts will be made to clearly document the outcome.
CRFs will consist of four-part NCR paper.
All CRFs will be completed in a neat, legible manner to ensure adequate interpretation of data. A black ballpoint pen will be used to ensure clarity of all reproduced CRFs.

15.3 Changes to CRF data

Errors occurring in CRFs will be crossed out with a single line without obscuring the initial entry, the correction will be written alongside the initial entry and the change will be initialed and dated by the investigator or designee. If not obvious, the reason of the change will be given on the additional comments pages. Correction fluid or any other means of obliteration of entries must not be used. Any correction made on the original CRF page must also appear clearly on all CRF pages copies.
When changes to CRF data are necessary following the removal of an original CRF from the study site, such changes will be documented on a query form that will be signed by the investigator or designee.

15.4 Provision of additional information

On request, the investigator will provide Transgene with additional data relating to the study, or copies of relevant source documents, duly anonymized. This is important when CRFs are illegible or when errors in data transcription are encountered. In case of particular issues or governmental queries, it may be necessary to have access to the complete source documents, provided that the patients' confidentiality is protected in accordance with applicable requirements.

16 REPORTING AND PUBLICATION

16.1 Clinical study report

All relevant data will be reported in a study report which will be prepared by Transgene and submitted for comments and signature to the study coordinator. The final report may be used for regulatory purposes as considered necessary by Transgene.

16.2 Confidentiality of study data

Any confidential information relating to the study drug or the study, including any data and results from the study is the property of Transgene. Documents are supplied to the investigators under conditions of strict confidentiality. Neither the investigator nor any person working on his/her behalf may disclose any of the information therein without having obtained prior written consent from Transgene.

16.3 Publication policy

The results of this study may be published or presented at scientific meetings. If this is envisaged, the investigators agree to submit all manuscripts or abstracts to Transgene prior to scientific meeting or journal submission. This allows Transgene to protect proprietary information and to provide comments based on information from other studies that may not yet be available to the investigator. The publication rules will follow the recruitment rate of evaluable patient: best recruiter-first name, the following names according to recruitment rate in the study, then Transgene representatives and study coordinator as the last name.
In accordance with consistent editorial practice, Transgene supports publication of multicenter studies in their entirety and not as individual center data unless ancillary study/data. A publication in which the contribution of Transgene's personnel exceeded that of conventional monitoring will be considered as a joint publication by the investigator and this person.

17 ARCHIVING

17.1 Investigator site file

The investigator is responsible for maintaining all the records that enable the conduct of the study at the site to be fully documented, in accordance with the ICH GCP standard.
This documentation will be kept by the investigator at least 2 years following the date the last marketing application is approved for the drug for the indication for which it is being investigated or if no application is to be filed or if the application is not approved for such indication, until 2 years after the investigation is discontinued. If longer archiving period is required, the local law/regulation must be followed. Transgene will inform the investigator when documents may be destroyed.
No study site document may be destroyed without prior written agreement between the investigator and Transgene. Should the investigator elect to assign the study documentation to another party, or move it to another location, Transgene must be notified.
If the investigator cannot guarantee this archiving requirement on site for any or all of the documents, special arrangements must be made between the investigator and Transgene to store these in a sealed container away from the site so it can be returned sealed to the investigator in case of an audit/inspection.

17.2 Trial master file

Transgene will archive the trial master file in accordance with GCP and applicable regulatory requirements and will inform the investigator when the study documentation can be destroyed.

18 REFERENCES

1. Willemze R, Jaffe ES, Burg G *et al*. WHO-EORTC classification for cutaneous lymphomas. Blood 2005; 105(10):3768-85.

2. Quesada JR, Kurzrock R, Sherwin SA, Gutterman JU. Phase II studies of recombinant human interferon gamma in metastatic renal cell carcinoma. J Biol Response Mod 1987; 6(1):20-7.

3. Garnick MB, Reich SD, Maxwell B, Coval-Goldsmith S, Richie JP, Rudnick SA. Phase I/II study of recombinant interferon gamma in advanced renal cell carcinoma. J Urol 1988; 139(2):251-5.

4. Creagan ET, Schaid DJ, Ahmann DL, Frytak S. Recombinant interferons in the management of advanced malignant melanoma. Updated review of five prospective clinical trials and long-term responders. Am J Clin Oncol 1988; 11(6):652-9.

5. Windbichler GH, Hausmaninger H, Stummvoll W *et al*. Interferon-gamma in the first-line therapy of ovarian cancer: a randomized phase III trial. Br J Cancer 2000; 82(6):1138-44.

6. Hilleman MR, Stallones RA, Gauld RL, Warfield MS, Anderson SA. Prevention of acute respiratory illness in recruits by adenovirus (RI-APC-ARD) vaccine. Proc Soc Exp Biol Med 1956; 92(2):377-83.

7. Simon R. Optimal two-stage designs for phase II clinical trials. Control Clin Trials 1989; 10(1):1-10.

8. Dummer R. Tumor-Plaque-Patch-Index as an evaluation proposal. Eur J Dermatol 1991; (1):135-8.

9. Lewis V, Finlay AY. 10 years experience of the Dermatology Life Quality Index (DLQI). J Investig Dermatol Symp Proc 2004; 9(2):169-80.

10. Finlay AY, Khan GK. Dermatology Life Quality Index (DLQI)--a simple practical measure for routine clinical use. Clin Exp Dermatol 1994; 19(3):210-6.

11. Tsanev R, Ivanov I. Biological activities of interferon gamma. Immune Interferon: Properties and Clinical Applications. Boca raton (Fla.): CRC Press, 2002: 25-37.

19 APPENDICES

Appendix A: Flow-Chart of Visits and Procedures

	Baseline[a]	Cycle 1				Cycle 2				Cycle 3				Cycle 4				End of Treatment Visit[b]	Follow-up Visit [c]
		Day 1	Day 8	Day 15	Day 29[de]	Day 1[d]	Day 8	Day 15	Day 29[de]	Day 1[d]	Day 8	Day 15	Day 29[de]	Day 1[d]	Day 8	Day 15	Day 29[e]		
TG1042 injection		X	X	X		X	X	X		X	X	X		X	X	X			
Complete medical history	X																		
Interim history		X	X	X	X[f]	X	X	X	X[f]	X	X	X	X[f]	X	X	X	X	X	X
Physical examination	X	X	X	X	X[f]	X	X	X	X[f]	X	X	X	X[f]	X	X	X	X	X	X
Vital signs	X	X	X	X	X[f]	X	X	X	X[f]	X	X	X	X[f]	X	X	X	X	X	X
Safety assessment		X	X	X	X[f]	X	X	X	X[f]	X	X	X	X[f]	X	X	X	X	X	
Tumor evaluation and imaging	X				X				X				X				X		X
Dermatology Life Quality Index	X				X				X				X				X		
Complete blood count	X	X	X	X	X[f]	X	X	X	X[f]	X	X	X	X[f]	X	X	X	X		
Standard laboratory parameters	X				X				X				X				X		
Pregnancy test	X																		
Proteomic & transcriptomic analysis	X																		
ECG	X				X[f]				X[f]				X[f]				X[f]		
CT or PET scan or PET/CTscan	X				X[g]				X[g]				X[g]				X[g]		
Bone marrow biopsy	X[hi]																		
Biopsy of CBCL lesion	X[i]				X[g]				X[g]				X[g]				X[g]		
Biopsy of peripheral lymph nodes	X[j]				X[j]				X[j]				X[j]				X[j]		

[a] To be performed within the 2 weeks preceding the 1st administration (except for biopsies).
[b] To be performed 4 weeks after the Day 29 of the last cycle.
[c] To be performed only for patients who completed the period of treatment without progression on a monthly basis during the first 6th months and then on a quarterly basis for 1 year.
[d] In case of continuation of treatment Day 1 of the next cycle and Day 29 of the previous cycle is the same day.
[e] To be performed also in case of withdrawal occurring during the cycle of treatment.
[f] To be performed only at the last cycle.
[g] To be performed only at the last cycle in case of complete response.
[h] If not previously performed since relapse.
[i] Within the 4 weeks preceding the 1st injection.
[j] To be performed only in case of suspicion of tumoral involvment.

Appendix B: Dermatology Life Quality Index[9]

The aim of this questionnaire is to measure how much your skin problem has affected your life OVER THE LAST WEEK. Please tick one box for each question.

1.	Over the last week, how **itchy, sore, painful** or **stinging** has your skin been?	Very much 3<br>A lot 2<br>A little 1<br>Not at all 0	
2.	Over the last week, how **embarrassed** or **self conscious** have you been because of your skin?	Very much 3<br>A lot 2<br>A little 1<br>Not at all 0	
3.	Over the last week, how much has your skin interfered with you going **shopping** or looking after your **home** or **garden?**	Very much 3<br>A lot 2<br>A little 1<br>Not at all 0	Not relevant
4.	Over the last week, how much has your skin influenced the **clothes** you wear?	Very much 3<br>A lot 2<br>A little 1<br>Not at all 0	Not relevant
5.	Over the last week, how much has your skin affected any **social** or **leisure** activities?	Very much 3<br>A lot 2<br>A little 1<br>Not at all 0	Not relevant
6.	Over the last week, how much has your skin made it difficult for you to do any **sport**?	Very much 3<br>A lot 2<br>A little 1<br>Not at all 0	Not relevant
7.	Over the last week, has your skin prevented you from **working** or **studying**?<br>If "No", over the last week how much has your skin been a problem at **work** or **studying**?	Yes 3<br>No<br>A lot 2<br>A little 1<br>Not at all 0	Not relevant
8.	Over the last week, how much as your skin created problems with your **partner** or any of your **close friends** or **relatives**?	Very much 3<br>A lot 2<br>A little 1<br>Not at all 0	Not relevant
9.	Over the last week, how much has your skin caused any **sexual difficulties**?	Very much 3<br>A lot 2<br>A little 1<br>Not at all 0	Not relevant
10.	Over the last week, how much a problem has the **treatment** for your skin been, for example by making your home messy, or by taking up time?	Very much 3<br>A lot 2<br>A little 1<br>Not at all 0	Not relevant

Please check you have answered EVERY question. Thank you. **Sum:**

Appendix C: Photographic Procedure

Objective

The goal is to take 4 images per area involved:
1 A framing image – about ½ body size.
2 An anatomic part image showing the anatomic part involved.
3 A close-up image as close to having the lesion fills the frame as possible.
4 A close-up 45-degree image. Fill the frame at 45 degrees.

Dress codes

Remove all clothing and jewellery except undergarment, unless those area are also involved.

Required materials

- Digital camera with minimal 800x600 resolution and a macro capability with zoom lens 35-105 mm (35 mm) equivalent.
- Storage card.
- Personal Computer (PC).
- Measurement tape.
- Skin marker.

Imaging

- Use one compact flash card per patient.
- Maintain automatic series number of images.
- Do not delete photos on the media while taking photos.
- Compact flash card is reused after the images are uploaded and deleted from card.

Lighting

- Background should be as uniform as possible.
- Whenever possible, a medium shade blue or grey drape as a background.
- Use a naturally wall lit room.
- Try to avoid intense bright, direct light and fluorescent light if possible.
- Use the camera's intrinsic flash.
- Do not use extra external sources of lighting.
- Always avoid causing a white-out due a too close shot.
- Avoid shots that are "back lit" (e.g. patient in front of bright window or against bright background). The goal is to avoid extremes of dark and light in a frame to maintain the correct exposure.
- Avoid "clutter" backgrounds that tend to be distracting.
- The flash option is set on automatic.

- Except is when taking scalp shots of patients with dark brown or black hair. A flash should not be used in this instance).

Focusing

- Use Auto focus.
- Place the area of interest in the center of the frame.
- Ensure that the picture is in focus prior to capturing image.
- Ensure that the macro selection button is selected for close distance.

Shooting

The goal is to take standardized images:

- Distant view to identify and localize all lesions.
- Medium view of each lesion showing the anatomic part involved.
- A close-up image of each lesion for measuring the lesion.

Distant view:

- Total body (head to toe) front.
- Total body (head to toe) back.

Medium view:

- Shot at 2 or more feet.

Close Up

- Take a close up image at the maximum optical zoom at the closest allowed distance.
- Close-up as close to having the lesion fill the frame as possible.
- The closest lesion for taking any photograph is 6 inches.
- Photograph each lesion at 90 degree angle to plane of the lesion.
- A centimeter ruler should be included in all close-up images.

Identification

Each image should be save as .mpeg format and identified as follows (each code will be separated by “#”):

- 3 digits for centre number.
- 3 digits for patient number.
- 3 digits for visit code (BAS for the baseline visit, Cxx for the visit of xx cycle, Fxx for the xxth follow-up visit).
- 2 digits for lesion number (AN for the total body front, PO for the total body back, XX for the lesion number xx).
- 2 digits for image number (01 for the medium distant image, 02 for the 90° close-up image and 03 for the 45° close-up image).

Storage

- Upload to secure hard drive with limited access.
- All images should be stored in a specific folder (one folder per patient and one sub-folder per visit).
- Open the image file and view the captured image using an imaging software and ensure the image is adequate.
- Ensure that the lesion is centered, marked and focused.
- Ensure that the lesion seen on the image is representative of the actual lesions on the patient. If not, recapture the image.
- No enhancement is made to the stored images.
- Copy the folder on a CD-rom labelling with the center number, the patient number and initials and the visit code.

Appendix D: Inclusion Procedure

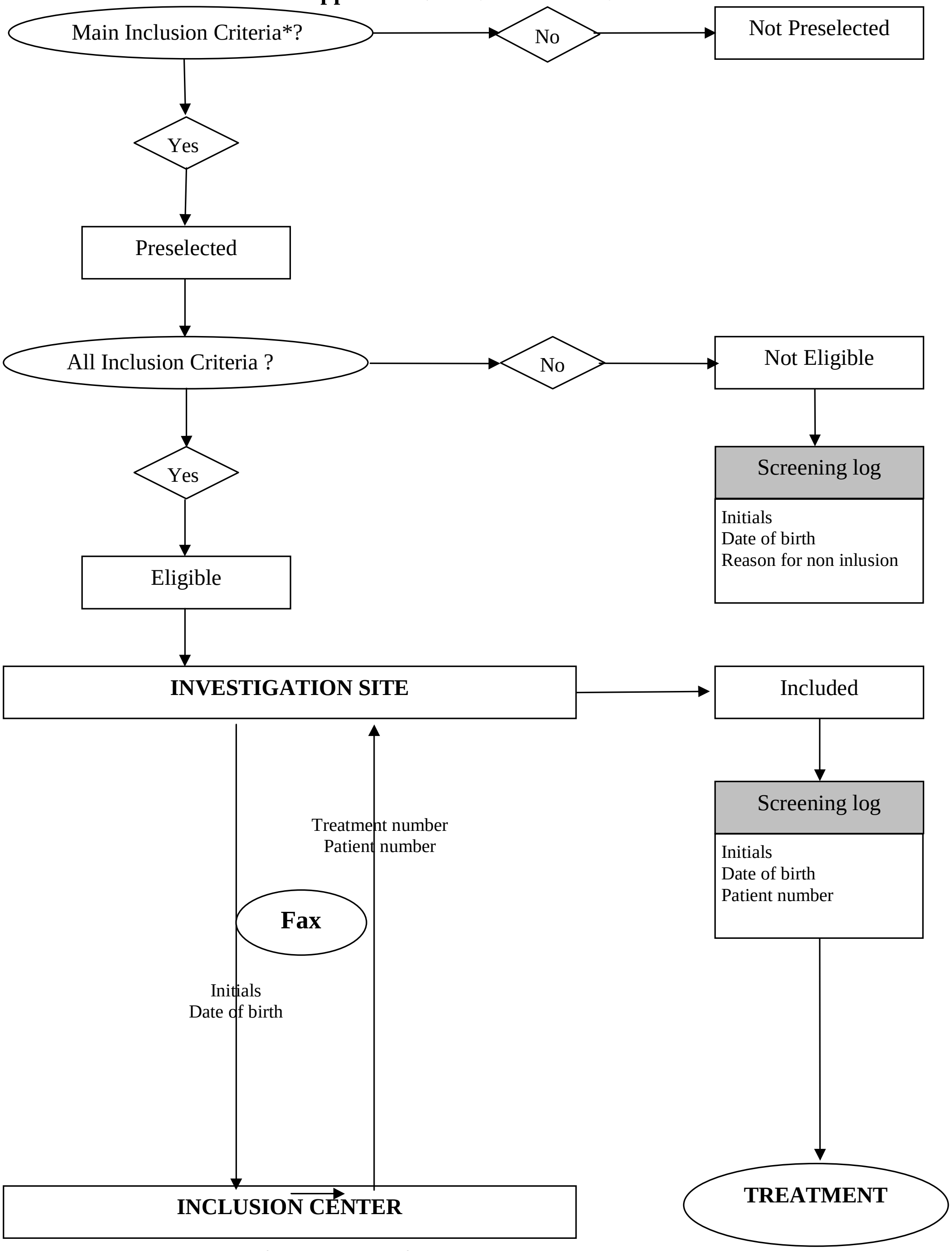

* Main inclusion criteria: History of CBCL, relapse after radiotherapy.

Appendix E: Performance Status (ECOG Scale)

0. Fully active, able to carry on all pre-disease performance without restriction.
1. Restricted in physically strenuous activity but ambulatory and able to carry out work of a light or sedentary nature, e.g. light house work, office work.
2. Ambulatory and capable of self care but unable to carry out any work activities. Up and about more than 50% of waking hours.
3. Capable of only limited self-care, confined to bed or chair more than 50% of waking hours.
4. Completely disabled. Cannot carry out any self-care. Totally confined to bed or chair.
